# Reorganization of synchronous and asynchronous release in developing hippocampal autaptic neurons of mice

**DOI:** 10.14814/phy2.71062

**Published:** 2026-08-17

**Authors:** Kouya Uchino, Hiroyuki Kawano, Risako Fujikawa, Takuya Watanabe, Katsunori Iwasaki, Shin‐Ya Kawaguchi, Shutaro Katsurabayashi

**Affiliations:** ^1^ Department of Neuropharmacology, Faculty of Pharmaceutical Sciences Fukuoka University Fukuoka Japan; ^2^ Department of Biophysics, Graduate School of Science Kyoto University Kyoto Japan; ^3^ Present address: Laboratory of Neural Membrane Biology, Graduate School of Brain Science Doshisha University Kyotanabe‐shi Kyoto Japan

**Keywords:** asynchronous release, synaptic development, synaptic transmission, synchronous release, vesicle pool dynamics

## Abstract

Presynaptic vesicle pools, particularly the readily releasable pool (RRP), are essential for synaptic transmission. The RRP comprises fast‐release pool (FRP) and slow‐release pool (SRP) subpopulations that mediate synchronous and asynchronous neurotransmitter release, respectively, yet their developmental regulation remains unclear. To investigate this question, we utilized a hippocampal autaptic culture system to analyze synaptic function in individual excitatory neurons at 1, 2, and 3 weeks in vitro. We observed significant enhancements in both synaptic transmission efficiency and the size of RRP with neuronal maturation. Despite the increase in vesicle availability, the probability of vesicular release (Pvr) decreased during development, suggesting a shift in RRP composition or priming status. Although both synchronous and asynchronous release increased in absolute magnitude during development, the relative contribution of asynchronous release to total release declined with maturation, indicating a shift toward synchronous release in mature neurons. Furthermore, while the overall RRP refilling rate remained constant across development, early recovery of EPSCs after depletion was modestly faster in mature neurons. This finding may suggest preferential recruitment of vesicles into the FRP in mature neurons. These findings demonstrate a developmental reorganization of presynaptic vesicle pools that optimizes synchronous release and enhances temporal precision in mature synapses.

## 1 INTRODUCTION

Synapses are specialized neuronal structures that mediate inter‐neuronal communication via neurotransmitter release. Synaptic vesicles loaded with neurotransmitters translocate to presynaptic active zones, where they engage with docking and priming proteins to form the readily releasable pool (RRP) (Südhof, 2012). However, vesicles within the RRP are not functionally homogeneous: they dynamically shift among primed states (Neher, 2024; Neher & Brose, 2018). These vesicles are classified into fast‐release pool (FRP) and slow‐release pool (SRP), according to their release kinetics, and cooperate to regulate neurotransmitter release (Sakaba & Neher, 2001). This functional segregation underlies two major release modes: synchronous release, tightly coupled to action potentials, and asynchronous release, triggered by residual Ca^2+^ accumulation during repetitive firing (Atwood & Karunanithi, 2002; Goda & Stevens, 1994; Otsu et al., 2004). In the calyx of Held synapse, brief Ca^2+^ influx primarily recruits FRP for synchronous release, whereas SRP activation drives asynchronous release under high‐frequency stimulation via residual calcium (Neher, 2017; Sakaba, 2006). Consistently, residual Ca^2+^ persisting after an action potential can evoke asynchronous vesicle fusion (Atluri & Regehr, 1998), and under high‐frequency stimulation, synchronous release declines while asynchronous release becomes dominant (Hagler Jr & Goda, 2001).

During synaptic development, neurons enhance the stability and efficiency of synaptic transmission through homeostatic plasticity in response to changes in synaptic input strength, affecting excitability (Shepherd et al., 2006; Wierenga et al., 2005), morphology (Harms & Craig, 2005; Kirov & Harris, 1999), synapse formation (McKinney et al., 1999; Moulder et al., 2006), and transmission efficacy (Abbott & Nelson, 2000; Davis, 2006; Murthy et al., 2001; Rich & Wenner, 2007; Turrigiano et al., 1998; Turrigiano & Nelson, 2004). The balance between synchronous and asynchronous release critically shapes postsynaptic spike probability and temporal precision, thereby contributing to stable and efficient synaptic transmission (Atwood & Karunanithi, 2002; Deng et al., 2020; Hefft & Jonas, 2005; Li et al., 2021; Luo & Südhof, 2017; Otsu et al., 2004). However, how this balance is developmentally regulated remains unclear.

To address this question, we examined how neuronal maturation affects the balance between synchronous and asynchronous release. To isolate cell‐autonomous mechanisms, we used single excitatory hippocampal neurons grown in autaptic culture on astrocytic microdots, thereby eliminating the network inputs present in mass‐dissociated or slice cultures (Bekkers & Stevens, 1991). This preparation provides a useful platform for examining developmental changes in synchronous and asynchronous release under well‐controlled conditions (Guzikowski et al., 2026). We specifically analyzed developmental changes in RRP size, vesicular release probability, release mode, and vesicle replenishment. Our aim was to determine how maturation reorganizes presynaptic vesicle pools to shape the stability and temporal precision of synaptic transmission.

## 2 METHODS

### 2.1 Animals

Timed‐pregnant Jcl:ICR mice (Catalog ID: Jcl:ICR, CLEA Japan, Inc., Tokyo, Japan) were obtained from the Kyudo Company (Tosu, Japan) at gestational Day 13. Pregnant Jcl:ICR mice, aged 15–17 weeks, were used in the study. They were housed in plastic cages under controlled conditions, with a room temperature of 23°C ± 2°C, a humidity level of 60% ± 2%, and a 12‐h light–dark cycle (lights on at 7:00 am and off at 7:00 pm). Food (CLEA Rodent Diet, CE‐2, CLEA Japan, Inc., Tokyo, Japan) and water were available ad libitum. The body weights of the pregnant mice were not recorded.

### 2.2 Cell culture

Astrocytes and neurons from newborn Jcl:ICR mice were cultured following previously described methods (Bekkers & Stevens, 1991; Oyabu et al., 2019). Briefly, cerebral cortices were isolated from newborn mice of either sex at postnatal Days 0–1, then enzymatically dissociated using trypsin. The resulting cells were cultured in 75 cm^2^ culture flasks (Cat. No. 430641; Corning Inc., Corning, NY, USA) in Dulbecco's Modified Eagle Medium with GlutaMAX (DMEM with GlutaMAX; Cat. No. 10569‐044; Thermo Fisher Scientific, Waltham, MA, USA) supplemented with 10% fetal bovine serum (FBS; Cat. No. 10437‐028; Thermo Fisher Scientific), 1% penicillin–streptomycin (Cat. No. 15140‐122; Thermo Fisher Scientific), and 0.1% MITO+ Serum Extender (Cat. No. 355006; Corning Inc.). After 2 weeks, non‐astrocytic cells were removed by tapping the culture flask multiple times. The astrocytes were then isolated and seeded at a density of 6000 cells/cm^2^ per well onto 22‐mm coverslips (thickness no. 1; Matsunami, Osaka, Japan) placed in 6‐well plates (Cat. No. 92406; TPP, Switzerland). The coverslips were pre‐coated with 0.5% agarose and subsequently stamped with a 1:1 mixture of rat‐tail collagen (final concentration 1.0 mg/mL, Cat. No. 354236; BD Biosciences, San Jose, CA, USA) and poly‐D‐lysine (final concentration 0.25 mg/mL, Cat. No. P6407; Sigma‐Aldrich, St. Louis, MO, USA) in 300‐μm square islands.

One week later, neurons were obtained from the hippocampus of another newborn mouse of either sex at postnatal Days 0–1. The dissociated neurons were plated at a density of 1500 cells/cm^2^ per well onto the micro‐island astrocytes and maintained in Neurobasal‐A Medium (Cat. No. 10888‐022; Thermo Fisher Scientific) supplemented with 2% B‐27 Supplement (Cat. No. 17504‐044; Thermo Fisher Scientific), 1% GlutaMAX Supplement (Cat. No. 35050‐061; Thermo Fisher Scientific), and 1% penicillin–streptomycin. The cultures were maintained in a humidified incubator at 37°C with 5% CO_2_ for 1 week, 2 weeks, and 3 weeks before being used for electrophysiological experiments. No medium exchange was performed during neuron culture.

### 2.3 Electrophysiological recording

Micro islands containing single neurons forming recurrent synapses (autapses) were used in this study. Data from three groups (1, 2, and 3 weeks in vitro) were collected from the same set of sister cultures. Synaptic responses were recorded in whole‐cell configuration under the voltage‐clamp condition (MultiClamp 700B, Molecular Devices, Union City, CA) at room temperature (approximately 23°C ± 2°C). The resistance of the patch pipette ranged from 2 to 5 MΩ, with 70%–90% of the access resistance compensated. Autaptic neurons exhibited evoked synaptic transmission in response to an action potential triggered by a brief (2 ms) somatic depolarization pulse to 0 mV via the patch pipette. Synaptic responses were recorded at a sampling rate of 20 kHz and filtered at 10 kHz. Data were excluded from analysis if the leak current exceeded 300 pA.

All data were analyzed offline using AxoGraph X 1.2 software (AxoGraph Scientific, Berkeley, CA, USA). The evoked EPSCs were confirmed to be mediated by the excitatory neurotransmitter glutamate, as they were effectively blocked by 6‐cyano‐7‐nitroquinoxaline‐2,3‐dione (CNQX) (data not shown).

During 20 Hz stimulation, synchronous and asynchronous release components were quantified as described previously (Otsu et al., 2004). Briefly, total release charge was obtained by integrating the current relative to the baseline measured immediately before the first stimulus of the train. Synchronous release charge was estimated by integrating the current relative to the local baseline measured immediately before each pulse. Asynchronous release charge was calculated by subtracting synchronous charge from the total release charge. The current was confirmed to return to baseline after the stimulation train, and recordings with unstable baseline were excluded from analysis.

### 2.4 Solutions and drug applications

The standard extracellular recording solution contained (in mM): NaCl 140, KCl 2.4, HEPES 10, glucose 10, CaCl_2_ 2, MgCl_2_ 1; pH 7.4. Patch pipettes were filled with internal solution (in mM): K‐gluconate 125, NaCl 10, MgCl_2_ 4.6, ATP‐Na_2_ 4, GTP‐Na_2_ 0.3, creatine phosphate 15 (Cat. No. P7936; Thermo Fisher Scientific), phosphocreatine kinase 20 U/mL (Cat. No. C3755; Thermo Fisher Scientific), EGTA 1; pH 7.4. To determine the size of the readily releasable pool (RRP) of synaptic vesicles, a hypertonic solution was prepared by adding sucrose to the standard extracellular solution to a final concentration of 0.5 M. Extracellular solutions were delivered via a fast‐flow application system (SF‐77B, Warner Instruments, Hamden, CT, USA). Each flow pipe had a large diameter (430 μm), ensuring uniform application of the solution to all regions of an autaptic neuron cultured on a 300 × 300 μm astrocytic microisland. This setup was well‐suited for sucrose application to elicit synaptic responses from all nerve terminals of the recorded neuron. Unless otherwise specified, all chemicals were obtained from Merck (Sigma‐Aldrich) (St. Louis, MO, USA).

### 2.5 Computational modeling

We constructed a mathematical model to simulate synaptic vesicle release from two distinct pools: FRP and SRP. The dynamics of these pools were governed by recovery rates (rec) and release probabilities (*p*). The simulation was performed with a time step of dt = 0.0001 s.

The synchronous releases upon the *i*‐th action potential during 20 Hz train (Rel_FRP_[*i*]) were defined by the following equation:

(1) $${\mathrm{Rel}}_{\mathrm{FRP}}\left(i\right)=\mathrm{FRP}\left(t\right)\times \mathrm{Pvr}$$

where FRP(*t*) and Pvr represent the number of vesicles in FRP at time *t* and the release probability of individual vesicles in FRP, respectively.

The asynchronous release at time *t* (Rel_SRP_[*t*]) was modeled as a function of the bulk Ca^2+^ concentration near the SRP vesicles (Ca_cyto_), following a Hill equation:

(2) $${\mathrm{Rel}}_{\mathrm{SRP}}\left(t\right)=\mathrm{SRP}\left(t\right)\times \alpha \cdot {\left(1+{\left(\frac{{\mathrm{Ca}}_{50}}{{\mathrm{Ca}}_{\text{cyto}}}\right)}^{n}\right)}^{-1}$$

where *α*, Ca_50_ and *n* represents the relative sensitivity of SRP vesicles for Ca^2+^, Ca^2+^ level causing half‐maximal release (10 μM) and Hill coefficient for Ca^2+^‐dependent release (3.5), respectively (Schneggenburger & Neher, 2000). Ca_cyto_ was assumed to increase instantaneously by Ca_influx_ (0.75 μM/AP) upon APs and subsequently decay exponentially depending on the leak constant Ca_leak_ (2/s).

To investigate the maturation of synaptic transmission, we compared two parameter sets:

1 week: Lower pool sizes (FRP = 1500, SRP = 2000) with higher synchronous release probability (Pvr = 0.06), and the rates vesicular replenishment (v[rec]_FRP_ = v[rec]_SRP_ = 0.21/s).

2, 3 weeks: Larger pool sizes (FRP = 6000, SRP = 5000) with a significant reduction in probability both for synchronous (Pvr = 0.018) and asynchronous (*α* = 0.55) releases, compared with those in 1 week. The rate for vesicular replenishment from the reserve pool was assumed to be slightly higher (0.24/s). The replenishment of the RRP, consisting of FRP and SRP, was calculated as the recovery process starting from a fully depleted state (FRP = 0, SRP = 0), in the model described above, assuming that the recovery of each pool during dt follows first‐order kinetics defined by the following equations:

(3) $$\frac{\text{dFRP}\left(t\right)}{\mathrm{dt}}=v{\left[\mathrm{rec}\right]}_{\mathrm{FRP}}\times \left({\mathrm{FRP}}_{\max}-\mathrm{FRP}\left(t\right)\right)$$

(4) $$\frac{\text{dSRP}\left(t\right)}{\mathrm{dt}}=v{\left[\mathrm{rec}\right]}_{\mathrm{SRP}}\times \left({\mathrm{SRP}}_{\max}-\mathrm{SRP}\left(t\right)\right)$$

where FRP_max_ and SRP_max_ represent the total FRP and SRP pool sizes, respectively, with distinct values for 1 and 2, 3 weeks. The relative recovery was calculated as the instantaneous pool size normalized by its maximum capacity to compare the recruitment efficiency across developmental stages.

The above equations were numerically solved by the Euler method with a time step (dt) of 0.0001 s using the Python programming language. The dt value was set so as to minimize the calculation error. The Python scripts used (Files S1 and S2) and the calculated data (File S3) are available as Supplementary files online.

### 2.6 Data analysis and statistics

Data are expressed as mean ± SD. *n* represents the number of cells recorded, and *N* represents the number of independent cell culture batches. Data from the three developmental groups were obtained from the same set of sister cultures. Normality of data distribution was assessed using the Shapiro–Wilk test. When normality assumptions were not met, comparisons among groups were performed using the Kruskal–Wallis test followed by Dunn's multiple comparisons test. Otherwise, comparisons among the three developmental groups were performed using one‐way ANOVA followed by Tukey's multiple comparisons test using Prism 10.4.0 software (GraphPad, La Jolla, CA, USA). Differences were considered significant when *p* < 0.05.

## 3 RESULTS

### 3.1 Developmental changes in RRP size and vesicular release probability

To examine developmental changes in synaptic transmission, we recorded autaptic cultures from neonatal ICR mice at 1 week (7–9 DIV), 2 weeks (13–15 DIV), and 3 weeks (21–27 DIV) under the voltage‐clamp conditions.

**FIGURE 1:**
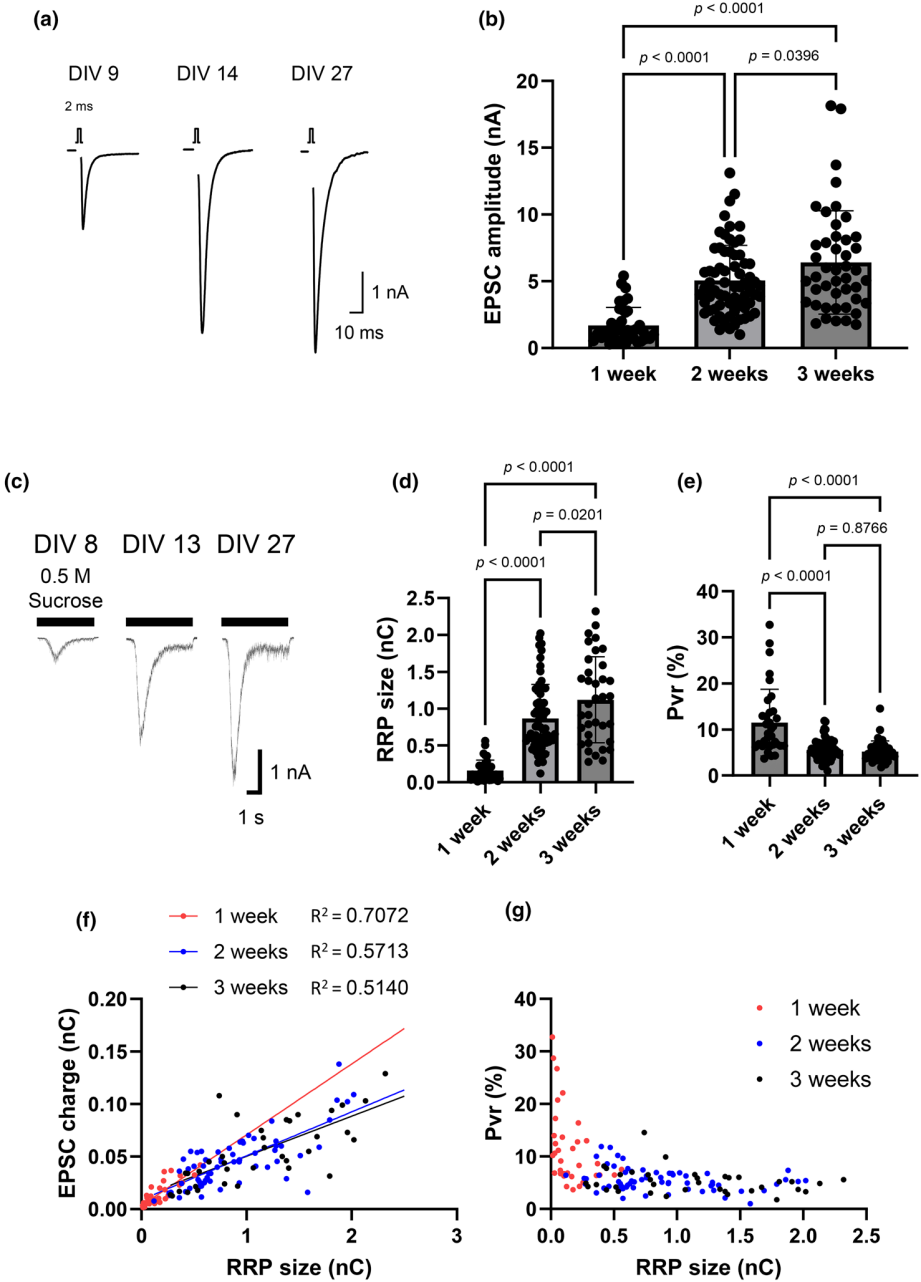
Developmental changes in RRP size and vesicle release probability. (a) Representative traces of EPSCs recorded from an autaptic neuron cultured for 9, 14, and 27 days in vitro (DIV). (b) Mean peak amplitudes of EPSCs at 1, 2, and 3 weeks in culture. (c) Representative traces of responses to hypertonic 0.5 M sucrose recorded from an autaptic neuron cultured for 8, 13, and 27 days in vitro (DIV). The bar above each trace indicates the duration of sucrose application. (d) Mean charge transfer in response to sucrose, representing the readily releasable pool (RRP) size. (e) Mean vesicle release probability (Pvr), calculated as the ratio of evoked EPSC charge to RRP charge in individual cells. (f) Scatter plot of readily releasable pool (RRP) size (*x*‐axis) versus EPSC charge (*y*‐axis) in the same cells at 1, 2, and 3 weeks in culture. Lines indicate linear regression fits. (g) Scatter plot of RRP size (*x*‐axis) versus the corresponding vesicular release probability (Pvr, %) (*y*‐axis) at 1, 2, and 3 weeks in culture. Data are presented as mean ± SD. Comparisons in (e) were made using the Kruskal–Wallis test followed by Dunn's multiple comparisons test. Other comparisons were made using one‐way ANOVA followed by Tukey's multiple comparisons test.

We then focused on AMPA receptor‐mediated excitatory postsynaptic currents (EPSCs). In isolated autaptic neurons, EPSC amplitudes following 2 ms voltage‐step–evoked Na^+^ currents increased progressively with culture age (1 week: 1.697 ± 1.346 nA, *n* = 38, *N* = 4; 2 weeks: 5.057 ± 2.631 nA, *n* = 67, *N* = 4; 3 weeks: 6.402 ± 3.866 nA, *n* = 45, *N* = 4; 1 vs. 2 weeks: *p* < 0.0001; 1 vs. 3 weeks: *p* < 0.0001; 2 vs. 3 weeks: *p* = 0.0396; Figure 1a,b).

Changes in EPSCs may reflect alterations in readily releasable pool (RRP) size or vesicular release probability (Basu et al., 2007; Rhee et al., 2005). To assess this, we applied 0.5 M sucrose for 5 s to deplete the RRP and quantified cumulative charge to estimate RRP size (Figure 1c) (Pyott & Rosenmund, 2002). The RRP size increased in a developmental stage–dependent manner (1 week: 0.1576 ± 0.1425 nC, *n* = 34, *N* = 4; 2 weeks: 0.8684 ± 0.4612 nC, *n* = 64, *N* = 4; 3 weeks: 1.120 ± 0.5818 nC, *n* = 36, *N* = 4; 1 vs. 2 weeks: *p* < 0.0001; 1 vs. 3 weeks: *p* < 0.0001; 2 vs. 3 weeks: *p* = 0.0201; Figure 1c,d).

Vesicular release probability (Pvr), defined as the evoked EPSC charge divided by the RRP charge, was highest at 1 week and roughly halved by 2 weeks, remaining stable thereafter (1 week: 11.50% ± 7.259%, *n* = 34, *N* = 4; 2 weeks: 5.549% ± 2.162%, *n* = 64, *N* = 4; 3 weeks: 5.202% ± 2.349%, *n* = 36, *N* = 4; 1 vs. 2 weeks: *p* < 0.0001; 1 vs. 3 weeks: *p* < 0.0001; 2 vs. 3 weeks: *p* = 0.8766; Figure 1e). These results indicate that, although RRP size increased during development, the release probability of individual vesicles decreased. It was unexpected that Pvr decreased as neurons developed. We therefore plotted the EPSC charge (*y*‐axis) against the RRP size of the same cell (*x*‐axis) at 1, 2, and 3 weeks to confirm the relationship between synaptic parameters (Figure 1f). This scatter plot showed that EPSC charge increased in parallel with RRP size. Linear regression analysis showed that the slope of this relationship was lower at 2 and 3 weeks than at 1 week (1 week: 0.06747, 95% CI 0.05184–0.08310; 2 weeks: 0.04181, 95% CI 0.03261–0.05100; 3 weeks: 0.03808, 95% CI 0.02517–0.05098). Next, we plotted Pvr on the *y*‐axis against the corresponding RRP size on the *x*‐axis (Figure 1g). Pvr exhibited an overall inverse relationship with RRP size. In particular, at 1 week, cells with smaller RRP sizes showed greater variability in Pvr. This heterogeneity likely contributes to the significant variation in Pvr values observed at 1 week in Figure 1e, indicating that immature neurons with small vesicle pools exhibit considerable variability in Pvr.

### 3.2 Developmental shift in the balance between synchronous and asynchronous release

**FIGURE 2:**
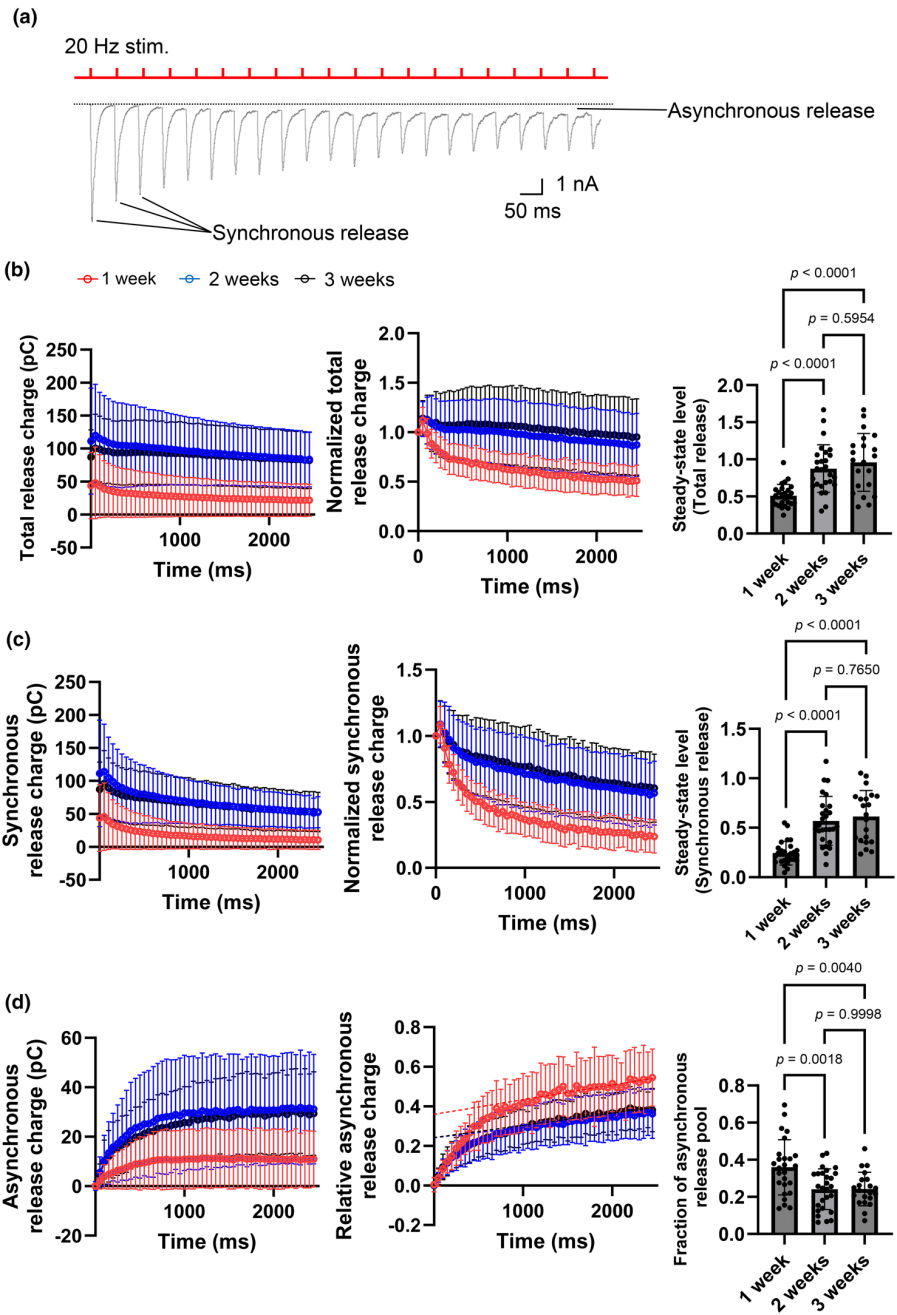
Developmental shift in the balance between synchronous and asynchronous release. (a) Representative EPSC trace during 20 Hz stimulation in an autaptic neuron. (b) Left, total release charge during 20 Hz stimulation. Middle, normalized total release charge during 20 Hz stimulation. Right, steady‐state level of normalized total release charge, calculated as the mean of the last five pulses in the 20 Hz stimulus train. (c) Left, synchronous release charge during 20 Hz stimulation, calculated by subtracting asynchronous release charge from total release charge. Middle, normalized synchronous release charge during 20 Hz stimulation. Right, steady‐state level of normalized synchronous release charge, calculated as the mean of the last five pulses in the 20 Hz stimulus train. (d) Left, asynchronous release charge during 20 Hz stimulation. Middle, relative asynchronous release charge during 20 Hz stimulation. The dashed line indicates a tangent fitted to the steady‐state phase, and the y‐intercept of this tangent was used as an estimate of the fraction of the asynchronous release pool. Right, fraction of the asynchronous release pool, obtained from the y‐intercept of a tangent fitted to the steady‐state phase of normalized asynchronous release. Data are presented as mean ± SD. Comparisons were made using one‐way ANOVA followed by Tukey's multiple comparisons test.

Evoked synaptic transmission comprises synchronous and asynchronous components, driven by action potential–evoked Ca^2+^ influx and residual Ca^2+^ accumulation, respectively (Chavis & Westbrook, 2001; Goda & Stevens, 1994). Despite the increase in the RRP from 1 to 2 weeks, we observed a paradoxical decrease in Pvr (Figure 1e). We hypothesized that this reflects a developmental reduction in the fraction of RRP vesicles belonging to the fast‐release pool (FRP), which mediates synchronous release. To test this, we applied a 20 Hz stimulation protocol for 2.5 s in autaptic neurons to assess the dynamics of synchronous and asynchronous release (Figure 2a). As stimulation progressed, EPSCs amplitude declined while asynchronous release increased. The total release charge (synchronous + asynchronous releases) was markedly greater at ≥2 weeks compared to 1 week (Figure 2b, left). Normalized total release charge, calculated relative to the first value, showed the most rapid decay during repeated stimulation at 1 week, whereas neurons at 2 and 3 weeks maintained more stable release (Figure 2b, middle). The average of the last five normalized total release charges in the 20 Hz stimulus train was used to assess steady‐state depression. As a result, the steady‐state level of normalized total release charge was greater after 2 weeks, indicating enhanced presynaptic reliability (1 week: 0.5112 ± 0.1521, *n* = 26, *N* = 3; 2 weeks: 0.8751 ± 0.3204, *n* = 27, *N* = 3; 3 weeks: 0.9595 ± 0.3900, *n* = 21, *N* = 3; 1 vs. 2 weeks: *p* < 0.0001; 1 vs. 3 weeks: *p* < 0.0001; 2 vs. 3 weeks: *p* = 0.5954; Figure 2b, right).

The synchronous release component was estimated by subtracting the asynchronous charge from the total release charge during individual 20 Hz stimulations. The synchronous component exhibited significantly greater charge accumulation after 2 weeks (Figure 2c, left). Similar to the total release charge, analysis of normalized synchronous charge (relative to the first value) showed the most rapid decay at 1 week, whereas neurons at 2 and 3 weeks maintained more stable release during repeated stimulation, indicating a developmental strengthening of synchronous release (Figure 2c, middle). Accordingly, the steady‐state level of normalized synchronous release, calculated as the average of the last five charges, was elevated after 2 weeks (1 week: 0.2441 ± 0.1198, *n* = 26, *N* = 3; 2 weeks: 0.5692 ± 0.2469, *n* = 27, *N* = 3; 3 weeks: 0.6131 ± 0.2621, *n* = 21, *N* = 3; 1 vs. 2 weeks: *p* < 0.0001; 1 vs. 3 weeks: *p* < 0.0001; 2 vs. 3 weeks: *p* = 0.7650; Figure 2c, right).

Next, the asynchronous release component was estimated by subtracting the synchronous charge from the total release charge during individual 20 Hz stimulations. Although the asynchronous charge also increased with age (Figure 2d, left), its proportion of the total evoked charge, namely, relative asynchronous release charge, was highest at 1 week and declined thereafter (Figure 2d, middle). The RRP has traditionally been estimated by extrapolating the steady‐state phase of cumulative EPSCs during an action potential train back to time zero (Schneggenburger et al., 1999). Using this same approach, we extrapolated the steady‐state phase of relative asynchronous release, and the *y*‐intercept was taken as the fraction of the asynchronous pool (Figure 2d, middle). This value was significantly reduced at 2 and 3 weeks compared with 1 week (1 week: 0.3595 ± 0.2621, *n* = 26, *N* = 3; 2 weeks: 0.2414 ± 0.1096, *n* = 27, *N* = 3; 3 weeks: 0.2422 ± 0.09018, *n* = 21, *N* = 3; 1 vs. 2 weeks: *p* = 0.0018; 1 vs. 3 weeks: *p* = 0.0040; 2 vs. 3 weeks: *p* = 0.9998; Figure 2d, right).

To further explore this relationship, we plotted the total release charge evoked by 20 Hz stimulation (*x*‐axis) against the fraction of the asynchronous release pool (*y*‐axis) (Figure S1A). No clear correlation was observed between these two parameters. However, the distribution of data points appeared to shift toward the lower right at 2 and 3 weeks compared with 1 week. These observations suggest that the relative contribution of the asynchronous release pool may decrease during maturation independently of total release charge, potentially reflecting a shift in the relative abundance of FRP versus SRP vesicles.

### 3.3 Vesicle replenishment kinetics are enhanced during development

Following vesicle fusion triggered by action potentials, reserve pool (RP) vesicles are recruited to replenish the RRP. Thus, during sustained stimulation, vesicle replenishment is actively engaged. To determine whether developmental changes in the synchronous/asynchronous balance are influenced by replenishment dynamics, we examined RRP refilling.

**FIGURE 3:**
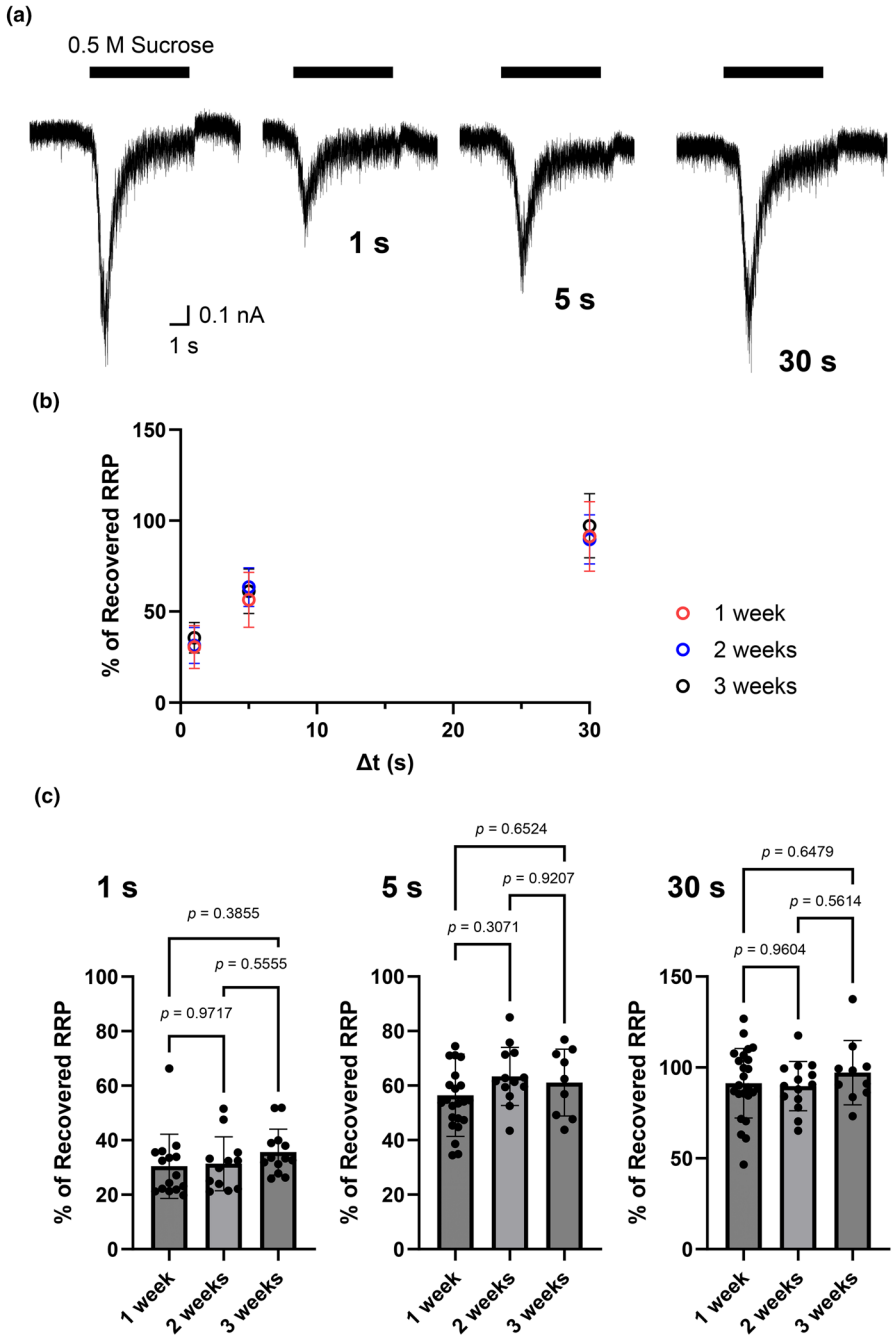
Developmental progression of RRP recovery rates. (a) Representative traces of synaptic responses to 0.5 M hypertonic sucrose applied at baseline and reapplied after 1, 5, and 30 s to assess recovery of the readily releasable pool (RRP). (b) Time course of RRP recovery, plotted as normalized charge relative to baseline (*x*‐axis, time after initial depletion; *y*‐axis, normalized recovery). (c) Bar graphs showing RRP recovery at 1 s (left), 5 s (middle), and 30 s (right) following initial sucrose depletion in autaptic neurons cultured for 1, 2, or 3 weeks. Data are presented as mean ± SD. Comparisons were made using one‐way ANOVA followed by Tukey's multiple comparisons test.

We first used repeated 0.5 M sucrose applications to deplete the RRP, and monitored recovery over time (Figure 3a). RRP recovery exhibited similar kinetics across all culture ages, indicating that the overall replenishment rate is not substantially altered during development (1 s: 1 week, 30.44% ± 11.80%, *n* = 15, *N* = 3; 2 weeks, 31.34% ± 9.903%, *n* = 12, *N* = 3; 3 weeks, 35.62% ± 8.449%, *n* = 13, *N* = 3; 1 vs. 2 weeks: *p* = 0.9717; 1 vs. 3 weeks: *p* = 0.3855; 2 vs. 3 weeks: *p* = 0.5555; 5 s: 1 week, 56.45% ± 15.11%, *n* = 23, *N* = 3; 2 weeks, 63.39% ± 10.66%, *n* = 13, *N* = 3; 3 weeks, 61.13% ± 12.25%, *n* = 9, *N* = 3; 1 vs. 2 weeks: *p* = 0.3071; 1 vs. 3 weeks: *p* = 0.6524; 2 vs. 3 weeks: *p* = 0.9207; 30 s: 1 week, 91.33% ± 19.14%, *n* = 24, *N* = 3; 2 weeks, 89.75% ± 13.53%, *n* = 14, *N* = 3; 3 weeks, 97.17% ± 17.67%, *n* = 10, *N* = 3; 1 vs. 2 weeks: *p* = 0.9604; 1 vs. 3 weeks: *p* = 0.6479; 2 vs. 3 weeks: *p* = 0.5614; Figure 3b,c).

**FIGURE 4:**
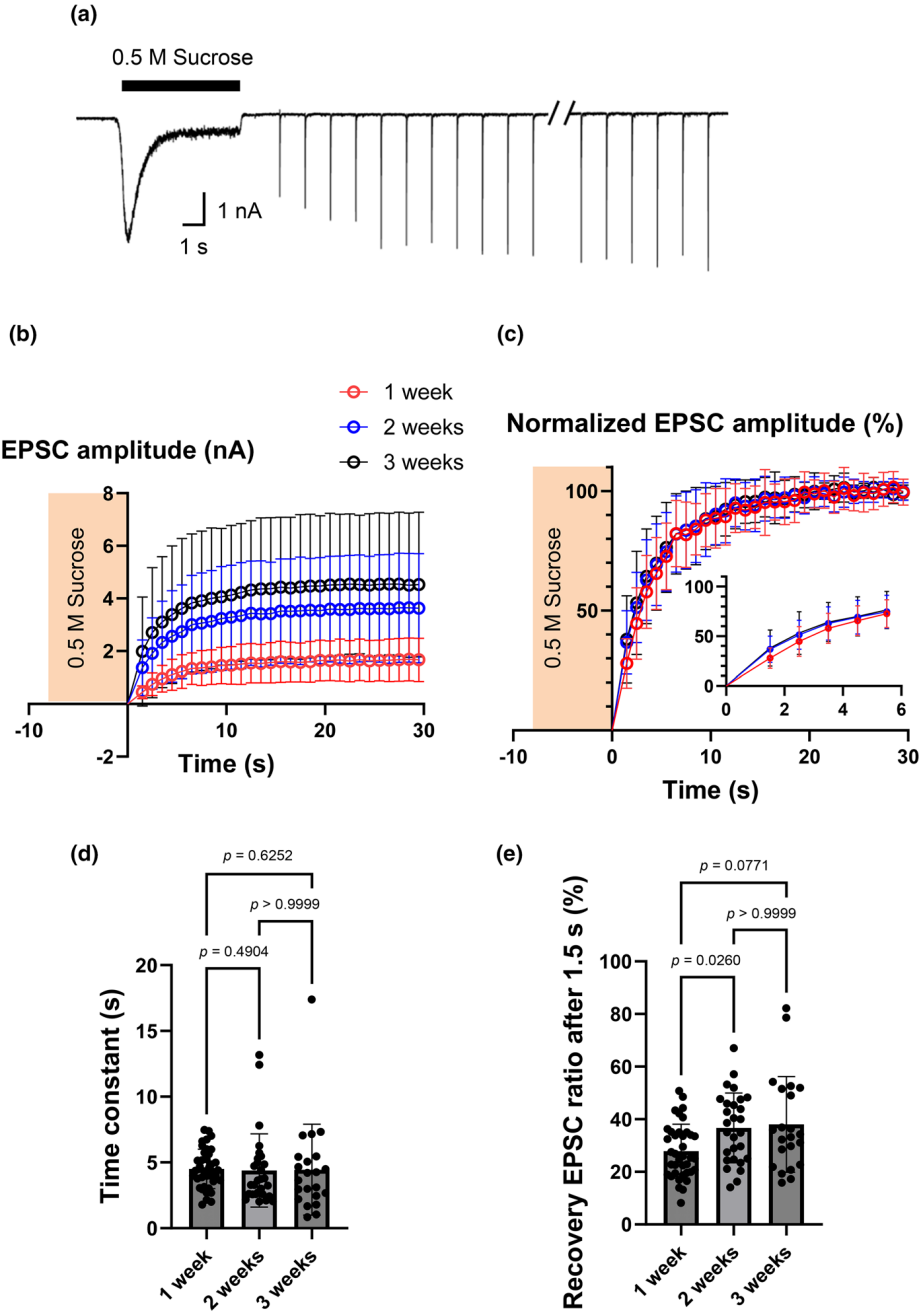
Developmental progression of EPSC recovery following RRP depletion. (a) Representative traces of synaptic responses evoked by 0.5 M hypertonic sucrose applied at baseline, followed by assessment of EPSC recovery over a 30 s interval. (b) Time course of EPSC amplitude recovery (*x*‐axis, time after initial depletion; *y*‐axis, absolute EPSC amplitude). (c) Time course of normalized EPSC recovery, with EPSC amplitudes normalized to the mean value at 25–30 s (*x*‐axis, time after initial depletion; *y*‐axis, normalized EPSC recovery). The inset shows an expanded view of the first five stimuli. (d) Bar graph showing the recovery time constants of EPSCs following removal of hypertonic sucrose in autaptic neurons cultured for 1, 2, or 3 weeks. Values were obtained from exponential fits to the data shown in (c). (e) Bar graphs showing EPSC recovery at 1.5 s after initial sucrose depletion in autaptic neurons cultured for 1, 2, or 3 weeks. Data are presented as mean ± SD. Comparisons in (d) and (e) were made using the Kruskal–Wallis test followed by Dunn's multiple comparisons test.

We next examined the recovery of evoked EPSCs following RRP depletion. Temporary RRP depletion was induced by the application of 0.5 M sucrose (Figure 4a). By 30 s post‐depletion, EPSC amplitudes had stabilized across all age groups (Figure 4a,b). To compare the recovery rate of EPSCs across culture ages, EPSCs were normalized to the average EPSC amplitude at the steady‐state level between 25 and 30 s (Figure 4c). Recovery time constants of EPSCs were obtained by fitting with a single‐exponential function (Stevens & Tsujimoto, 1995). The time constants were similar across culture ages (1 week: 4.510 ± 1.498 s, *n* = 38, *N* = 3; 2 weeks: 4.393 ± 2.785 s, *n* = 28, *N* = 3; 3 weeks: 4.449 ± 3.454 s, *n* = 22, *N* = 3; 1 vs. 2 weeks: *p* = 0.4904; 1 vs. 3 weeks: *p* = 0.6252; 2 vs. 3 weeks: *p* > 0.9999; Figure 4d). However, at the early time point of 1.5 s after RRP depletion, EPSC recovery was significantly faster at 2 weeks than at 1 week, with a similar tendency at 3 weeks (1 week: 27.88 ± 10.28%, *n* = 38, *N* = 3; 2 weeks: 36.73 ± 13.26%, *n* = 28, *N* = 3; 3 weeks: 38.02 ± 18.16%, *n* = 22, *N* = 3; 1 vs. 2 weeks: *p* = 0.0260; 1 vs. 3 weeks: *p* = 0.0771; 2 vs. 3 weeks: *p* > 0.9999; Figure 4c,e).

To further explore this relationship, we plotted the recovery EPSC ratio at 1.5 s after RRP depletion against the RRP size measured by 0.5 M sucrose application (Figure S1B). No clear correlation was observed between these two parameters. However, the distribution of data points appeared to shift toward the upper right at 2 and 3 weeks compared with 1 week. These observations suggest that early EPSC recovery after depletion may change during maturation independently of RRP size. This difference was observed despite a developmental decrease in Pvr and may reflect a developmental change in vesicle recruitment after depletion.

### 3.4 Computational modeling of synaptic vesicle release dynamics

To examine the certainty of the above qualitatively suggested developmental changes in presynaptic vesicle pool sizes for FRP/SRP, their vesicular release probability, and replenishment kinetics, we constructed a simple mathematical kinetic model for simulation of dynamics of vesicular releases and recoveries (for detail, see Method). As suggested by the data shown in Figure 2, the model assumed distinct release profiles between the two developmental stages. First, the FRP/SRP pool sizes are assumed to increase (1500/2000 at 1 week and 6000/5000 at 2, 3 weeks), leading to the four‐fold increase in the total FRP size during development. Second, the release probability of individual FRP vesicles upon a single action potential was set 0.06 (1 week) and 0.018 (2, 3 weeks), which looks smaller than the actual values estimated in Figure 1e. However, because RRP estimates obtained with hypertonic sucrose may be influenced by desensitization of postsynaptic AMPA receptors, this factor may contribute to the apparent discrepancy between the experimentally estimated Pvr values and the values used in the model. In terms of asynchronous release from SRP vesicles, we simply estimated the course of release events based on the 3.5th power dependency of release on bulk Ca^2+^ level assuming half‐maximal activation of 10 μM (Kawaguchi & Sakaba, 2015; Schneggenburger & Neher, 2000). Because of the lack of information about the actual Ca^2+^ increase from the experiments here, we hypothetically calculated that the bulk Ca^2+^ concentration around the SRP vesicles, which were assumed homogeneously distributed in the pool, increases instantaneously upon an action potential (0.75 μM/AP), and monotonically decreases at a rate of 2/s. Third, the recovery kinetics for FRP and SRP (0.21/s) were defined based on the experimental data shown in Figure 3. We additionally tested a slightly faster recovery rate from 0.21 to 0.24/s.

**FIGURE 5:**
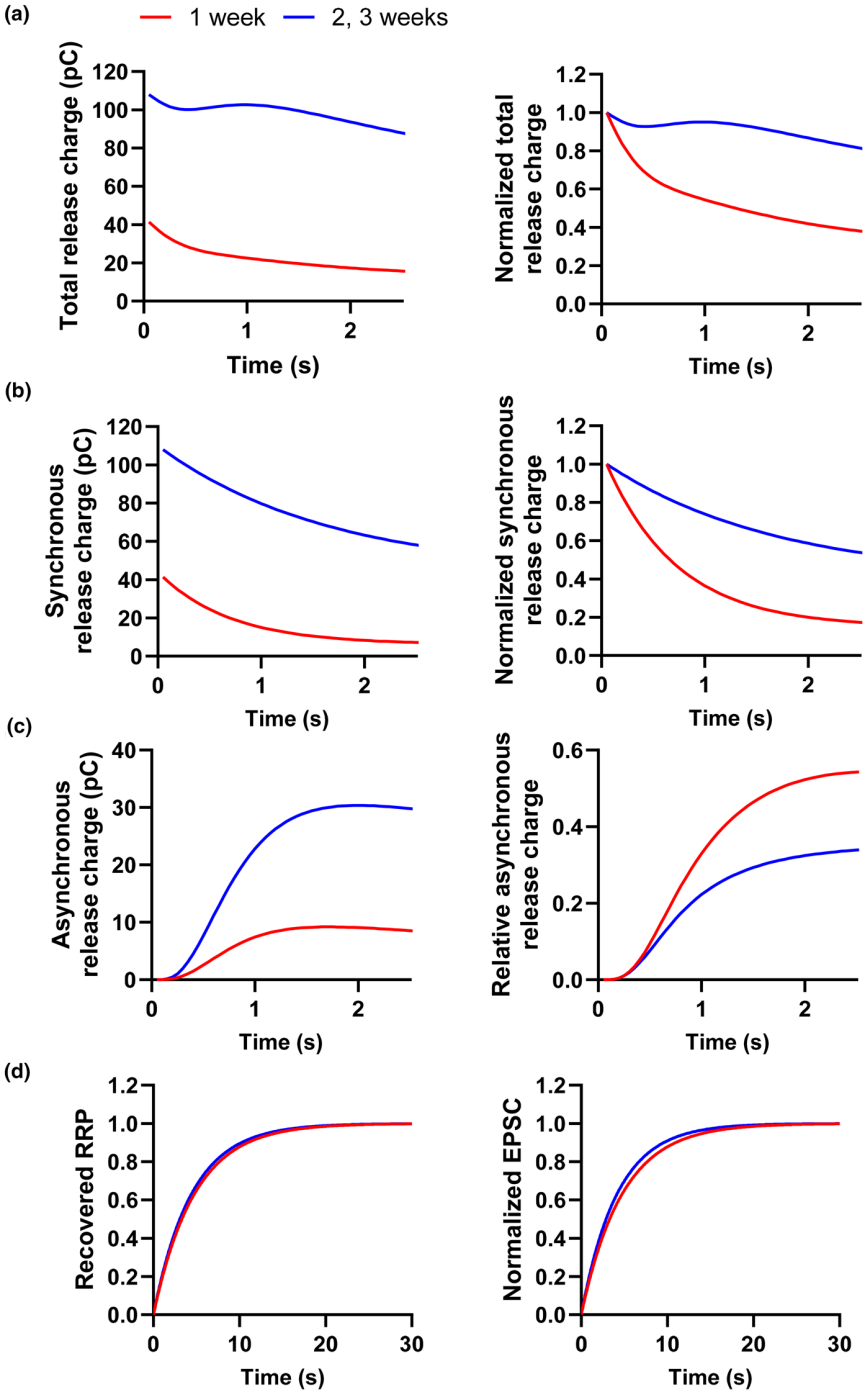
Numerical simulation of synaptic vesicle release and recovery from depletion. (a–c) Simulated time courses of total synaptic release (a), synchronous release from the fast‐releasing pool (FRP) (b), and asynchronous release from the slow‐releasing pool (SRP) (c) during 20 Hz repetitive stimulation, using the parameter sets described in Methods for 1 week (red) and 2 and 3 weeks (blue). Left panels show absolute values, whereas right panels show data normalized to the first response (a, b) or the fraction of asynchronous release relative to total release (c). (d) Relative recovery of the readily releasable pool (RRP) (left) and EPSC amplitude (right) from a fully depleted state.

Simulating the synaptic release changes during 20 Hz action potentials (experimentally tested in Figure 2), quicker stimulus‐dependent depression of synchronous releases, reflecting gradual depletion of FRP, was evident at 1 week (Figure 5b). On the other hand, the asynchronous release showed similar time courses during repetitive stimulation in both developmental ages (Figure 5c), although that in the simulation showed a slower initial rise compared to experimental observations (see Figure 2d). Nevertheless, the courses of total releases and relative contribution of asynchronous releases were quite similar to the experimental data (Figure 5a, compare with Figure 2b). Furthermore, the processes of vesicular replenishment after depletion, shown in Figures 3 and 4, were also qualitatively reproduced by the simulation (Figure 5d). The model also reproduced the modest difference in early recovery of synchronous release when a slightly faster replenishment rate was assigned to the FRP. Thus, the simulation was consistent with the possibility that developmental changes in vesicle recruitment contribute to early synchronous recovery. Remarkably, simulation analysis highlighted differences in the ratio SRP/RRP estimated from the extrapolation of experimental data (0.37 at 1 week; 0.21 at 2, 3 weeks) and that in model adjusted to best match the actual experimental release data (0.57 at 1 week; 0.45 at 2, 3 weeks). This discrepancy may reflect the difficulty in estimation of the contribution of a certain pool among a few pools, all of which are under the dynamic changes during repetitive activity. Taken together, our simple model simulation qualitatively and quantitatively consolidated the conclusions on developmental shifts in presynaptic release probability and vesicular dynamics.

## 4 DISCUSSION

### 4.1 Developmentally regulated synaptic maturation in a single‐neuron culture

Extensive studies over the past several decades have examined the developmental trajectory of synaptic function in cultured mammalian neurons. Variability in reported findings likely reflects differences in recording time points, tissue sources, culture conditions, and activity manipulations, as well as the involvement of multiple regulatory processes, including activity‐dependent homeostatic plasticity (Harms & Craig, 2005; Nakayama et al., 2005; Wierenga et al., 2005).

In the present study, we employed a single‐neuron autaptic culture system to minimize the complexity of network interactions and focus on cell‐autonomous mechanisms of synaptic development. This preparation enables precise evaluation of synaptic changes in the absence of extrinsic inputs. From 1 to 3 weeks in vitro, we observed marked developmental enhancements in excitatory synaptic transmission, indicating that maturation proceeds even in isolated neurons. These findings suggest that intrinsic activity and cell‐autonomous regulatory mechanisms can support functional synapse development without network‐level interactions.

### 4.2 Developmental changes in vesicle fusion probability and its underlying mechanisms

In this study, we observed that although the number of releasable vesicles—reflected by the readily releasable pool (RRP) size—increased after 2 weeks in culture, the vesicular release probability (Pvr) of evoked EPSCs paradoxically decreased (Figure 1). This apparent discrepancy may be explained by developmental changes in the molecular heterogeneity of synaptic vesicle pools, particularly in the relative proportion of vesicles competent for fast release. These observations led us to hypothesize that the proportion of fast‐release pool (FRP) vesicles responsible for synchronous release declines during maturation.

At 1 week, synchronous release exhibited a steep decay during 20 Hz stimulation, consistent with a higher initial Pvr (Figure 2c). Furthermore, the proportion of the asynchronous release pool was significantly reduced at 2 and 3 weeks compared with 1 week (Figure 2d). These findings indicate that the developmental decrease in Pvr cannot be attributed to changes in the relative contribution of asynchronous release.

One plausible mechanism underlying this shift is a developmental change in the molecular priming state of vesicles. Previous studies have distinguished between “loosely” and “tightly” docked and primed vesicle states (Neher & Brose, 2018) and have identified a subclass of highly fusogenic “super‐primed” vesicles characterized by rapid fusion kinetics. The abundance of super‐primed vesicles is regulated by presynaptic proteins such as Rab3 and Munc13 (Rhee et al., 2002; Schlüter et al., 2006). Thus, changes in the expression or activity of these molecules during development could alter the ratio of super‐primed to conventionally primed vesicles, leading to a decrease in overall Pvr despite an increased RRP size.

It is also possible that the absolute number of primed vesicles is lower at early developmental stages, resulting in a higher proportion of vesicles adopting a super‐primed state. This could account for the elevated Pvr observed at 1 week. Supporting this idea, even at 1 week, neurons with larger RRP sizes tended to show lower Pvr values (Figure 1g). Taken together, our findings suggest that developmental regulation of vesicle fusion probability is influenced not only by changes in vesicle pool size but also potentially by alterations in vesicle priming state and molecular composition.

### 4.3 Developmental reorganization of FRP and SRP

Analysis of synaptic responses during 20 Hz stimulation revealed that the relative contribution of asynchronous release declined markedly after 2 weeks in culture, while synchronous release became more dominant. This suggests that synaptic maturation involves both structural and functional reorganization of the RRP, specifically reflecting a shift in the relative composition of the FRP and SRP. These findings are consistent with previous observations at the calyx of Held synapse, where immature terminals exhibit prominent asynchronous release, whereas mature synapses favor synchronous release (Chuhma & Ohmori, 1998). In addition, in the somatosensory thalamus, the proportion of the fast component increases in mature synapses at surviving presynaptic terminals (Midorikawa & Miyata, 2021). This developmental increase in the proportion of the fast component observed in autaptic neurons in the present study may therefore reflect an integrated outcome of maturation‐related changes in surviving presynaptic terminals. Such developmental reorganization of vesicle pools may reflect a developmental shift toward greater reliance on synchronous transmission, thereby supporting a more reliable mode of information transfer in mature synapses.

**FIGURE 6:**
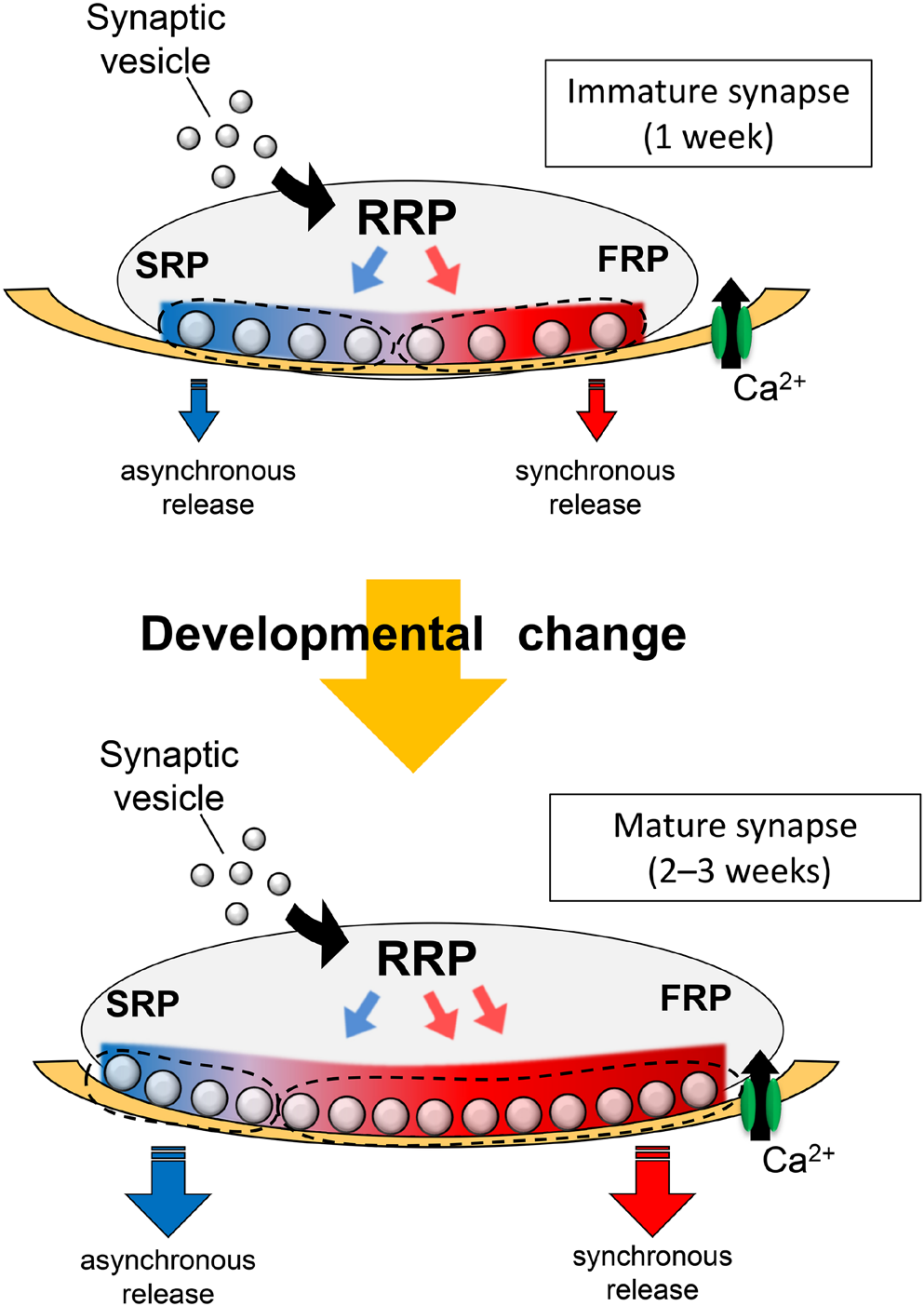
Proposed model of developmental changes in vesicle pool composition and dynamics. The readily releasable pool (RRP) of synaptic vesicles is functionally heterogeneous, comprising two distinct subpools: A fast‐release pool (FRP) and a slow‐release pool (SRP). Asynchronous release is thought to originate predominantly from the SRP, whereas synchronous release arises from vesicles with a high vesicular release probability (Pvr), primarily within the FRP. Based on our findings, we propose that the proportion of SRP vesicles may decrease during synaptic development. Although the overall rate of vesicle refilling into the RRP remains unchanged, newly recruited vesicles may be preferentially allocated to the FRP during synaptic development.

Although the overall rate of RRP refilling, as measured by sucrose‐evoked responses, did not differ significantly across developmental stages, early recovery of synchronous transmission after RRP depletion was modestly enhanced with maturation (Figures 3 and 4). This finding suggests that developmental changes in the molecular and spatial characteristics of vesicle refilling may influence the early phase of synchronous recovery (Figure 6). Several factors may underlie this preferential allocation, including developmental alterations in the spatial coupling between vesicles and Ca^2+^ channels, as well as isoform‐specific expression of synaptotagmins that differentially regulate vesicle fusion kinetics (Kaeser & Regehr, 2014; Midorikawa et al., 2024; Stevens & Sullivan, 2003; Wadel et al., 2007; Wu et al., 2024). It is important to note, however, that the dynamics of vesicle pool replenishment may depend on the stimulation protocol employed. For instance, the vesicle populations mobilized by hypertonic sucrose application may differ from those engaged during sustained high‐frequency stimulation (He et al., 2017; Jockusch et al., 2007; Junge et al., 2004; Lipstein et al., 2013; Wang & Kaczmarek, 1998; Weingarten et al., 2022). Therefore, future studies using more physiologically relevant stimulation paradigms will be required to dissect the precise mechanisms underlying FRP/SRP remodeling during synaptic development.

### 4.4 Computational modeling of vesicle release dynamics

In this study, we constructed a mathematical model to account for developmental changes in FRP and SRP sizes, release probability, and vesicle replenishment kinetics. The model indicates that the experimentally observed changes in the ratio of synchronous to asynchronous release and the release dynamics during repetitive stimulation can be reproduced by an increase in FRP and SRP sizes, a decrease in the release probability of FRP vesicles, and a modest acceleration of vesicle replenishment selectively to the FRP.

In this model, asynchronous release from SRP vesicles was described using a Ca^2+^‐dependent Hill function, assuming a homogeneous distribution of SRP vesicles. With this simplification, the time course of asynchronous release qualitatively agreed with the experimental results; however, the initial rise of asynchronous release was slower than that observed experimentally (Figure 5c). This discrepancy may be attributable to the assumption of SRP homogeneity. In actual synapses, a subpopulation of SRP vesicles located near Ca^2+^ channels or possessing higher Ca^2+^ sensitivity may exist heterogeneously (Neher, 2024) and may be released even at lower Ca^2+^ concentrations.

Although this effect was not prominent under the present experimental condition (20 Hz for 2.5 s), the model predicts that increasing the stimulation frequency or prolonging the stimulation duration would induce pronounced depression of asynchronous release from the SRP. Asynchronous release produces sustained synaptic transmission driven by residual Ca^2+^ and temporally integrates depolarization and Ca^2+^ elevation in postsynaptic neurons (Deng et al., 2020; Hefft & Jonas, 2005; Li et al., 2021). Therefore, suppression of excessive asynchronous release may help limit prolonged excitation and preserve the temporal precision of synaptic transmission.

Taken together, the present mathematical model provides a unified framework to explain developmental changes in the balance between synchronous and asynchronous release and the reorganization of vesicle replenishment dynamics. The selective increase in the replenishment rate of the FRP and the relative reduction in the contribution of the SRP may contribute to reliable synchronous transmission in mature synapses.

### 4.5 Comparison between in vitro developmental models and in vivo synaptic maturation

In vivo, the number of synapses rapidly increases after birth, peaking during adolescence before undergoing activity‐dependent pruning that refines neuronal circuits. This developmental trajectory is strongly shaped by sensory experiences and social interactions, and in adulthood, synaptic plasticity becomes largely experience‐dependent.

In the present study, we employed an autaptic culture system in which individual hippocampal neurons form self‐synapses in isolation from other neuronal inputs. We observed a robust enhancement of synaptic function during the first 2 weeks in vitro, followed by stabilization of synaptic properties by the third week. This temporal pattern of synaptic development and stabilization parallels in vivo transitions from synaptogenesis to circuit refinement. Thus, our findings suggest that the autaptic system, despite its simplicity, provides a valuable model for investigating the intrinsic, cell‐autonomous mechanisms underlying synaptic maturation and homeostatic regulation during early postnatal development.

## AUTHOR CONTRIBUTIONS

**Kouya Uchino:** Conceptualization; data curation; formal analysis; funding acquisition; investigation; methodology. **Hiroyuki Kawano:** Data curation; formal analysis; investigation; methodology. **Risako Fujikawa:** Supervision. **Takuya Watanabe:** Supervision. **Katsunori Iwasaki:** Supervision. **Shin‐Ya Kawaguchi:** Funding acquisition; investigation; methodology. **Shutaro Katsurabayashi:** Conceptualization; data curation; formal analysis; funding acquisition; investigation; methodology; project administration; resources; software; supervision; validation; visualization.

## FUNDING INFORMATION

This work was supported by a KAKENHI Grant‐in‐Aid for Transformative Research Areas (A) (No. 24H02336 to S.K., and 25H02611 to S.‐Y.K.), and a KAKENHI Grant‐in‐Aid for Scientific Research (B) (No. 23K27496 and 23K27575 to S.K., and 25K02362 to S.‐Y.K.), a KAKENHI Grant‐in‐Aid for Research Activity Start‐up (No. 25K23755 to K.U.), and a Research Grant from the Mochida Memorial Foundation for Medical and Pharmaceutical Research to K.U.

## CONFLICT OF INTEREST STATEMENT

The authors declare no conflicts of interest.

## ETHICS STATEMENT

All procedures related to animal care were carried out in accordance with the regulations set by the Fukuoka University Experimental Animal Welfare Committee, which aligns with NIH guidelines. The experiment was conducted only after receiving formal approval of the experimental protocol from the Committee (Approval No. 2311081 and 2510075). Cultured cells were obtained from neonatal mice euthanized by decapitation for tissue collection, and efforts were made to minimize distress.

## Supporting information


**Figure S1.** Relationships between release properties and RRP recovery. (A) Scatter plot of total release charge (*x*‐axis) versus the fraction of the asynchronous release pool (*y*‐axis) in the same cells at 1, 2, and 3 weeks in culture. (B) Scatter plot of RRP size (*x*‐axis) versus the EPSC recovery ratio at 1.5 s after RRP depletion (*y*‐axis) in the same cells at 1, 2, and 3 weeks in culture.

**File S1.** The Python scripts used for Computational modeling (Rel_model).

**File S2.** The Python scripts used for Computational modeling (Rel_model_rel_recover).

**File S3.** The calculated data for Computational modeling.

## Data Availability

All data that support the findings of this study are available from the corresponding author upon reasonable request.
