# Supplementary material for: Reorganization of synchronous and asynchronous release in developing hippocampal autaptic neurons of mice
Source: Physiol Rep. 2026 Aug 17;14(16):e71062. doi: 10.14814/phy2.71062 (PMC13481710; doi:10.14814/phy2.71062)
Supplement: Supplementary file 1 [file PHY2-14-e71062-s001.pdf]

```
# simple simulation code for vesicle releases in Uchino et al.
# running this python script provides a csv file "sim_rel_result.csv" in the same
directory.

import math
import matplotlib.pyplot as plt

# --- Parameter setting ---
dt = 0.0001
N_steps = 26000
Ca_in = 0.75
Ca_leak = 2
Ca_50 = 10

FRP_Week1, rec_f_1, p_f_1 = 1500, 0.21, 0.06
FRP_Week2, rec_f_2, p_f_2 = 6000, 0.24, 0.018
SRP_Week1, rec_s_1, p_s_1 = 2000, 0.21, 1
SRP_Week2, rec_s_2, p_s_2 = 5000, 0.21, 0.55

# --- Initial values ---
FRP_1, FRP_2 = FRP_Week1, FRP_Week2
SRP_1, SRP_2 = SRP_Week1, SRP_Week2
Ca_S, AP_time = 0.1, 0
S_rel_1, S_rel_2 = 0.0, 0.0
rel_f_1, rel_f_2 = 0.0, 0.0

# Data list
t_hist = []
relf1_hist, relf2_hist = [], []
cas_hist = []
S_rel1_hist, S_rel2_hist = [], []

# --- data listing ---
results = ["t, FRP_1, rel_f_1, FRP_2, rel_f_2, SRP_1, S_rel_1, SRP_2, S_rel_2, Ca_S"]
# Listing initial conditions
initial_line = f"0.0000, {FRP_1:.4f}, {rel_f_1:.4f}, {FRP_2:.4f}, {rel_f_2:.4f},
{SRP_1:.4f}, {S_rel_1:.4f}, {SRP_2:.4f}, {S_rel_2:.4f}, {Ca_S:.4f}"
results.append(initial_line)

# --- Main ---
for count in range(1, N_steps + 1):
    t = count * dt
    AP_time += 1

    if Ca_S > 0:
        Ca_S -= Ca_S * Ca_leak * dt
        if Ca_S < 0: Ca_S = 0.1

    # SRP dynamics
    rel_s_1 = SRP_1 * p_s_1 * dt * (1 / (1 + (Ca_50 / Ca_S)**3.5))
    rel_s_2 = SRP_2 * p_s_2 * dt * (1 / (1 + (Ca_50 / Ca_S)**3.5))
    SRP_1 += -rel_s_1 + rec_s_1 * dt * (SRP_Week1 - SRP_1)
    SRP_2 += -rel_s_2 + rec_s_2 * dt * (SRP_Week2 - SRP_2)
    S_rel_1 += rel_s_1
    S_rel_2 += rel_s_2

    # FRP recovery
    FRP_1 += rec_f_1 * dt * (FRP_Week1 - FRP_1)
    FRP_2 += rec_f_2 * dt * (FRP_Week2 - FRP_2)

    if AP_time == 500:
        # Relese from FRP upon an AP
        rel_f_1 = FRP_1 * p_f_1
```

```
        rel_f_2 = FRP_2 * p_f_2
        FRP_1 -= rel_f_1
        FRP_2 -= rel_f_2
        Ca_S += Ca_in

        # Data addition as strings
        line = f"{t:.4f}, {FRP_1:.4f}, {rel_f_1:.4f}, {FRP_2:.4f}, {rel_f_2:.4f}, 
{SRP_1:.4f}, {S_rel_1:.4f}, {SRP_2:.4f}, {S_rel_2:.4f}, {Ca_S:.4f}"
        results.append(line)

        t_hist.append(t)
        relf1_hist.append(rel_f_1)
        relf2_hist.append(rel_f_2)
        cas_hist.append(Ca_S)
        S_rel1_hist.append(S_rel_1)
        S_rel2_hist.append(S_rel_2)

        AP_time = 0
        S_rel_1, S_rel_2 = 0, 0

# --- Save data as csv file ---
with open("sim_rel_result.csv", mode="w") as f_out:
    f_out.write("¥n".join(results))

# --- Graphing ---
fig, (ax1, ax2, ax3) = plt.subplots(3, 1, figsize=(10, 10), sharex=True)

ax1.plot(t_hist, relf1_hist, 'o-', label='rel_f_1 (Week1)', color='red', markersize=4)
ax1.plot(t_hist, relf2_hist, 's-', label='rel_f_2 (Week2)', color='blue', markersize=4)
ax1.set_ylabel("Synchronous Release")
ax1.set_title("Synaptic Dynamics Simulation (FRP & SRP)")
ax1.legend(); ax1.grid(True)

ax2.plot(t_hist, S_rel1_hist, 'o-', label='S_rel_1', color='red', markersize=4)
ax2.plot(t_hist, S_rel2_hist, 's-', label='S_rel_2', color='blue', markersize=4)
ax2.set_ylabel("Asynchronous Release (SRP)")
ax2.legend(); ax2.grid(True)

ax3.plot(t_hist, cas_hist, color='red', label='Ca_S')
ax3.set_xlabel("Time (s)"); ax3.set_ylabel("Ca Concentration")
ax3.legend(); ax3.grid(True)

plt.tight_layout()
plt.show()

print("Simulation completed successfully.")
```
