# Supplementary material for: Reorganization of synchronous and asynchronous release in developing hippocampal autaptic neurons of mice
Source: Physiol Rep. 2026 Aug 17;14(16):e71062. doi: 10.14814/phy2.71062 (PMC13481710; doi:10.14814/phy2.71062)
Supplement: Supplementary file 2 [file PHY2-14-e71062-s002.pdf]

Supplementary file 2
(Rel_model_rel_recover)

```
# simple simulation code for vesicle releases in Uchino et al.
# running this python script provides a csv file "sim_Recover_result.csv" in the same directory.

import math
import matplotlib.pyplot as plt

# --- Parameter setting ---
dt = 0.0001
N_steps = 300000  # for 30sec sim

# Pool sizes and recovery kinetics
FRP_Week1, rec_f_1 = 1500, 0.21
FRP_Week2, rec_f_2 = 6000, 0.24
SRP_Week1, rec_s_1 = 2000, 0.21
SRP_Week2, rec_s_2 = 5000, 0.21

# RRP (FRP + SRP)
RRP_Week1 = FRP_Week1 + SRP_Week1
RRP_Week2 = FRP_Week2 + SRP_Week2

# --- Initial value (start from 0) ---
FRP_1, FRP_2 = 0.0, 0.0
SRP_1, SRP_2 = 0.0, 0.0
AP_time = 0

# Data list
t_hist = []
FRP_rel_1, FRP_rel_2 = [], []
SRP_rel_1, SRP_rel_2 = [], []
RRP_rel_1, RRP_rel_2 = [], []

# Header listing data sets
results = ["t, FRP_1, FRP_2, SRP_1, SRP_2"]

# --- Main ---
for count in range(1, N_steps + 1):
    t = count * dt
    AP_time += 1

    # FRP/SRP renewal (for individual time step)
    FRP_1 += rec_f_1 * dt * (FRP_Week1 - FRP_1)
    FRP_2 += rec_f_2 * dt * (FRP_Week2 - FRP_2)
    SRP_1 += rec_s_1 * dt * (SRP_Week1 - SRP_1)
    SRP_2 += rec_s_2 * dt * (SRP_Week2 - SRP_2)

    # data sampling each 500 steps (0.05 sec)
    if AP_time >= 500:
        t_hist.append(t)

        # calcluate relative recovery
        FRP_rel_1.append(FRP_1 / FRP_Week1)
        FRP_rel_2.append(FRP_2 / FRP_Week2)
        SRP_rel_1.append(SRP_1 / SRP_Week1)
        SRP_rel_2.append(SRP_2 / SRP_Week2)
        RRP_rel_1.append((FRP_1 + SRP_1) / RRP_Week1)
        RRP_rel_2.append((FRP_2 + SRP_2) / RRP_Week2)

        # data setting for saving
        line = f"{t:.4f}, {FRP_1:.4f}, {FRP_2:.4f}, {SRP_1:.4f}, {SRP_2:.4f}"
        results.append(line)

        AP_time = 0
```

```
# --- Data saving as csv --
with open("sim_Recover_result.csv", mode="w") as f_out:
    f_out.write("¥n".join(results))

# --- graphing ---
fig, (ax1, ax2) = plt.subplots(2, 1, figsize=(10, 8), sharex=True)

# Relative recovery of FRP
ax1.plot(t_hist, FRP_rel_1, label='FRP_1 (Week1)', color='red')
ax1.plot(t_hist, FRP_rel_2, label='FRP_2 (Week2)', color='blue')
ax1.set_ylabel("Relative Recovery (FRP)")
ax1.set_title("Normalized Pool Recovery Dynamics (No Calcium)")
ax1.set_ylim(0, 1.1)
ax1.legend()
ax1.grid(True)

# Relative recovery of total RRP
ax2.plot(t_hist, RRP_rel_1, label='RRP_1 Total', color='red', linewidth=2)
ax2.plot(t_hist, RRP_rel_2, label='RRP_2 Total', color='blue', linewidth=2)
ax2.set_ylabel("Relative Recovery (Total RRP)")
ax2.set_xlabel("Time (s)")
ax2.set_ylim(0, 1.1)
ax2.legend()
ax2.grid(True)

plt.tight_layout()
plt.show()

print("Simulation (No Calcium) completed.")
```
