# Supplementary material for: Reorganization of synchronous and asynchronous release in developing hippocampal autaptic neurons of mice
Source: Physiol Rep. 2026 Aug 17;14(16):e71062. doi: 10.14814/phy2.71062 (PMC13481710; doi:10.14814/phy2.71062)
Supplement: Supplementary file 3 [file PHY2-14-e71062-s003.pdf]

Supplementary file 3
(calculated data)

time (s)	FRP_1	rel_f_1	FRP_2	rel_f_2	SRP_1	S_rel_1	SRP_2	S_rel_2	Ca_S	Q_rel_f_1	Q_rel_f_2	rel_rel_f_1	rel_rel_f_2	Q_S_rel_1	Q_S_rel_2	tot_Q_rel_	tot_Q_rel_	rel_tot_rel	rel_tot_rel_2	rel_asy_1	rel_asy_2	0
0.05	1410	90	5892	108	2000	0	5000	0	0.759	41.4	108	1	1	0	0	41.4	108	1	1	0	0	
0.1	1326.284	84.6564	5787.209	106.0792	1999.99	0.0102	4999.986	0.014	1.4368	38.94194	106.0792	0.940627	0.982215	0.004692	0.014	38.94664	106.0932	0.94074	0.982344	0.00012	0.000132	
0.15	1248.412	79.6859	5685.532	104.2155	1999.896	0.0947	4999.857	0.1303	2.0501	36.65551	104.2155	0.885399	0.964958	0.043562	0.1303	36.69908	104.3458	0.886451	0.966165	0.001187	0.001249	
0.2	1175.978	75.0624	5586.876	102.4071	1999.571	0.3279	4999.41	0.4509	2.605	34.5287	102.4071	0.834027	0.948214	0.150834	0.4509	34.67954	102.858	0.83767	0.952389	0.004349	0.004384	
0.25	1108.601	70.7617	5491.151	100.6525	1998.825	0.7548	4998.384	1.0381	3.107	32.55038	100.6525	0.786241	0.931968	0.347208	1.0381	32.89759	101.6906	0.794628	0.94158	0.010554	0.010208	
0.3	1045.927	66.7613	5398.271	98.95	1997.456	1.3891	4996.5	1.9108	3.5613	30.7102	98.95	0.741792	0.916204	0.638986	1.9108	31.34918	100.8608	0.757227	0.933896	0.020383	0.018945	
0.35	987.63	63.0402	5308.151	97.2981	1995.276	2.2185	4993.501	3.0529	3.9724	28.99849	97.2981	0.700447	0.900908	1.02051	3.0529	30.019	100.351	0.725097	0.929176	0.033995	0.030422	
0.4	933.4029	59.5789	5220.708	95.6953	1992.129	3.2136	4989.168	4.425	4.3443	27.40629	95.6953	0.661988	0.886068	1.478256	4.425	28.88455	100.1203	0.697694	0.92704	0.051178	0.044197	
0.45	882.9619	56.3593	5135.864	94.1401	1987.9	4.3358	4983.339	5.9752	4.6809	25.92528	94.1401	0.626214	0.871668	1.994468	5.9752	27.91975	100.1153	0.67439	0.926994	0.071436	0.059683	
0.5	836.0425	53.3644	5053.541	92.6311	1982.513	5.5433	4975.908	7.6476	4.9854	24.54762	92.6311	0.592938	0.857695	2.549918	7.6476	27.09754	100.2787	0.65453	0.928506	0.094101	0.076263	
0.55	792.399	50.5787	4973.663	91.1669	1975.937	6.7961	4966.822	9.3887	5.2609	23.2662	91.1669	0.561986	0.844138	3.126206	9.3887	26.39241	100.5556	0.637498	0.93107	0.118451	0.093368	
0.6	751.8026	47.9874	4896.16	89.7463	1968.174	8.0587	4956.079	11.1512	5.5102	22.0742	89.7463	0.533193	0.830984	3.707002	11.1512	25.78121	100.8975	0.622734	0.934236	0.143787	0.11052	
0.65	714.0406	45.5771	4820.959	88.3679	1959.256	9.3015	4943.713	12.8954	5.7358	20.96547	88.3679	0.506412	0.818221	4.27869	12.8954	25.24416	101.2633	0.609762	0.937623	0.169492	0.127345	
0.7	678.9151	43.335	4747.993	87.0304	1949.239	10.501	4929.791	14.5901	5.9399	19.9341	87.0304	0.4815	0.805837	4.83046	14.5901	24.76456	101.6205	0.598178	0.940931	0.195055	0.143574	
0.75	646.242	41.2495	4677.194	85.7327	1938.193	11.64	4914.402	16.2119	6.1246	18.97477	85.7327	0.458328	0.793821	5.3544	16.2119	24.32917	101.9446	0.587661	0.943931	0.220081	0.159027	
0.8	615.8501	39.3096	4608.5	84.4735	1926.202	12.7064	4897.649	17.7446	6.2917	18.08242	84.4735	0.436773	0.782162	5.844944	17.7446	23.92736	102.2181	0.577956	0.946464	0.244279	0.173595	
0.85	587.5801	37.5051	4541.846	83.2518	1913.355	13.6927	4879.645	19.1775	6.4429	17.25235	83.2518	0.416723	0.77085	6.298642	19.1775	23.55099	102.4293	0.568864	0.948419	0.267447	0.187227	
0.9	561.2838	35.8266	4477.174	82.0663	1899.745	14.5951	4860.51	20.5051	6.5797	16.48024	82.0663	0.398073	0.759873	6.713746	20.5051	23.19398	102.5714	0.560241	0.949735	0.289461	0.199911	
0.95	536.8235	34.2653	4414.422	80.9161	1885.464	15.4126	4840.361	21.7253	6.7035	15.76204	80.9161	0.380726	0.749223	7.089796	21.7253	22.85183	102.6414	0.551977	0.950383	0.310251	0.211662	
1	514.071	32.813	4353.536	79.8	1870.602	16.1464	4819.314	22.8388	6.8156	15.09398	79.8	0.364589	0.738889	7.427344	22.8388	22.52132	102.6388	0.543993	0.950359	0.329792	0.222516	
1.05	492.9071	31.4622	4294.458	78.7172	1855.247	16.7992	4797.484	23.8486	6.9169	14.47261	78.7172	0.34958	0.728863	7.727632	23.8486	22.20024	102.5658	0.536238	0.949683	0.348088	0.23252	
1.1	473.2208	30.2056	4237.136	77.6665	1839.479	17.3749	4774.976	24.7589	7.0086	13.89458	77.6665	0.335618	0.719134	7.992454	24.7589	21.88703	102.4254	0.528672	0.948383	0.365169	0.241726	
1.15	454.9089	29.0367	4181.518	76.647	1823.376	17.8779	4751.891	25.575	7.0916	13.35688	76.647	0.32263	0.709694	8.223834	25.575	21.58072	102.222	0.521273	0.9465	0.381073	0.250191	
1.2	437.8756	27.9495	4127.552	75.6578	1807.008	18.3132	4728.324	26.3026	7.1667	12.85677	75.6578	0.31055	0.700535	8.424072	26.3026	21.28084	101.9604	0.51403	0.944078	0.395852	0.257969	
1.25	422.0315	26.9382	4075.189	74.698	1790.44	18.6859	4704.361	26.9479	7.2346	12.39157	74.698	0.299313	0.691648	8.595514	26.9479	20.98709	101.6459	0.506934	0.941166	0.409562	0.265115	
1.3	407.2936	25.9975	4024.382	73.7667	1773.731	19.0011	4680.082	27.517	7.2961	11.95885	73.7667	0.288861	0.683025	8.740506	27.517	20.69936	101.2837	0.499984	0.937812	0.42226	0.271682	
1.35	393.5847	25.1224	3975.085	72.8631	1756.936	19.264	4655.561	28.0159	7.3517	11.5563	72.8631	0.279138	0.674658	8.86144	28.0159	20.41774	100.879	0.493182	0.934065	0.434007	0.277718	
1.4	380.8329	24.3085	3927.253	71.9863	1740.102	19.4792	4630.864	28.4505	7.402	11.18191	71.9863	0.270094	0.66654	8.960432	28.4505	20.14234	100.4368	0.48653	0.92997	0.444856	0.283268	
1.45	368.9714	23.5514	3880.842	71.1356	1723.273	19.6513	4606.05	28.8265	7.4476	10.83364	71.1356	0.261682	0.658663	9.039598	28.8265	19.87324	99.9621	0.48003	0.925575	0.454863	0.288374	
1.5	357.9381	22.8471	3835.81	70.3102	1706.487	19.7848	4581.175	29.1491	7.4888	10.50967	70.3102	0.253857	0.65102	9.101008	29.1491	19.61067	99.4593	0.473688	0.920919	0.464084	0.293076	
1.55	347.6751	22.192	3792.116	69.5093	1689.778	19.8835	4556.287	29.4232	7.526	10.20832	69.5093	0.246578	0.643605	9.14641	29.4232	19.35473	98.9325	0.467506	0.916042	0.472567	0.297407	
1.6	338.1287	21.5827	3749.721	68.7321	1673.176	19.9512	4531.43	29.6536	7.5598	9.928042	68.7321	0.239808	0.636408	9.177552	29.6536	19.10559	98.3857	0.461488	0.910979	0.480359	0.301402	
1.65	329.2487	21.0159	3708.585	67.9781	1656.707	19.9913	4506.643	29.8444	7.5903	9.667314	67.9781	0.23351	0.629427	9.195998	29.8444	18.86331	97.8225	0.455636	0.905764	0.487507	0.305087	
1.7	320.9888	20.4886	3668.671	67.2465	1640.396	20.007	4481.96	29.9997	7.6179	9.424756	67.2465	0.227651	0.622653	9.20322	29.9997	18.62798	97.2462	0.449951	0.900428	0.494054	0.308492	
1.75	313.3056	19.9982	3629.944	66.5366	1624.26	20.001	4457.413	30.1229	7.6429	9.199172	66.5366	0.222202	0.61608	9.20046	30.1229	18.39963	96.6595	0.444436	0.894995	0.500035	0.311639	
1.8	306.1587	19.542	3592.367	65.8479	1608.318	19.9759	4433.027	30.2175	7.6655	8.98932	65.8479	0.217133	0.609703	9.188914	30.2175	18.17823	96.0654	0.439088	0.889494	0.50549	0.314551	
1.85	299.5109	19.1177	3555.907	65.1795	1592.583	19.9342	4408.828	30.2864	7.686	8.794142	65.1795	0.212419	0.603514	9.169732	30.2864	17.96387	95.4659	0.43391	0.883944	0.510454	0.317248	
1.9	293.3272	18.723	3520.53	64.5311	1577.07	19.8779	4384.836	30.3324	7.7045	8.61258	64.5311	0.208033	0.59751	9.143834	30.3324	17.75641	94.8635	0.428899	0.878366	0.514959	0.319748	
1.95	287.5753	18.3559	3486.204	63.9019	1561.786	19.8089	4361.069	30.3579	7.7212	8.443714	63.9019	0.203954	0.591684	9.112094	30.3579	17.55581	94.2598	0.424053	0.872776	0.519036	0.322066	
2	282.2249	18.0144	3452.898	63.2914	1546.742	19.7289	4337.543	30.3653	7.7364	8.286624	63.2914	0.20016	0.586031	9.075294	30.3653	17.36192	93.6567	0.41937	0.867192	0.522713	0.324219	
2.05	277.2481	17.6967	3420.582	62.6991	1531.944	19.6395	4314.272	30.3565	7.7501	8.140482	62.6991	0.19663	0.580547	9.03417	30.3565	17.17465	93.0556	0.414847	0.861626	0.526018	0.326219	
2.1	272.6187	17.4012	3389.226	62.1243	1517.398	19.542	4291.266	30.3334	7.7625	8.004552	62.1243	0.193347	0.575225	8.98932	30.3334	16.99387	92.4577	0.41048	0.85609	0.528974	0.328079	
2.15	268.3126	17.1263	3358.802	61.5666	1503.107	19.4377	4268.537	30.2977	7.7738	7.878098	61.5666	0.190292	0.570061	8.941342	30.2977	16.81944	91.8643	0.406267	0.850595	0.531608	0.329809	
2.2	264.3071	16.8707	3329.282	61.0255	1489.075	19.3277	4246.091	30.2509	7.7839	7.760522	61.0255	0.187452	0.565051	8.890742	30.2509	16.65126	91.2764	0.402204	0.845152	0.533938	0.331421	
2.25	260.5813	16.6329	3300.639	60.5005	1475.304	19.2129	4223.936	30.1943	7.7931	7.651134	60.5005	0.18481	0.56019	8.837934	30.1943	16.48907	90.6948	0.398287	0.839767	0.535987	0.332922	
2.3	257.1156	16.4116	3272.847	59.9911	1461.794	19.0942	4202.077	30.1292	7.8014	7.549336	59.9911	0.182351	0.555473	8.783332	30.1292	16.33267	90.1203	0.394509	0.834447	0.537777	0.334322	
2.35	253.8919	16.2059	3245.881	59.4968	1448.547	18.9724	4180.519	30.0566	7.8089	7.454714	59.4968	0.180066	0.550896	8.727304	30.0566	16.18202	89.5534	0.39087	0.829198	0.539321	0.335628	
2.4	250.8933	16.0145	3219.716	59.0172	1435.561	18.8482	4159.264	29.9776	7.8158	7.36667	59.0172	0.177939	0.546456	8.670172	29.9776	16.03684	88.9948	0.387363	0.824026	0.540641	0.336847	
2.45	248.104	15.8364	3194.328	58.5518	1422.837	18.7222	4138.316	29.893	7.8219	7.284744	58.5518	0.17596	0.542146	8.612212	29.893	15.89696	88.4448	0.383984	0.818933	0.541752	0.337985	
2.5	245.5094	15.6708	3169.695	58.1003	1410.372	18.5949	4117.675	29.8036	7.8275	7.208568	58.1003	0.17412	0.537966	8.553654	29.8036	15.76222	87.9039	0.38073	0.813925	0.542668	0.339048	
2.55	243.096	15.5168	3145.794	57.6622	1398.165	18.4667	4097.343	29.7101	7.8325	7.137728	57.6622	0.172409	0.533909	8.494682	29.7101	15.63241	87.3723	0.377594	0.809003	0.543402	0.34004	
2.6	240.8512	15.3735	3122.603	57.2371	1386.213	18.3382	4077.319	29.6132	7.8371	7.07181	57.2371	0.170817	0.529973	8.435572	29.6132	15.50738	86.8503	0.374574	0.804169	0.543971	0.340968	

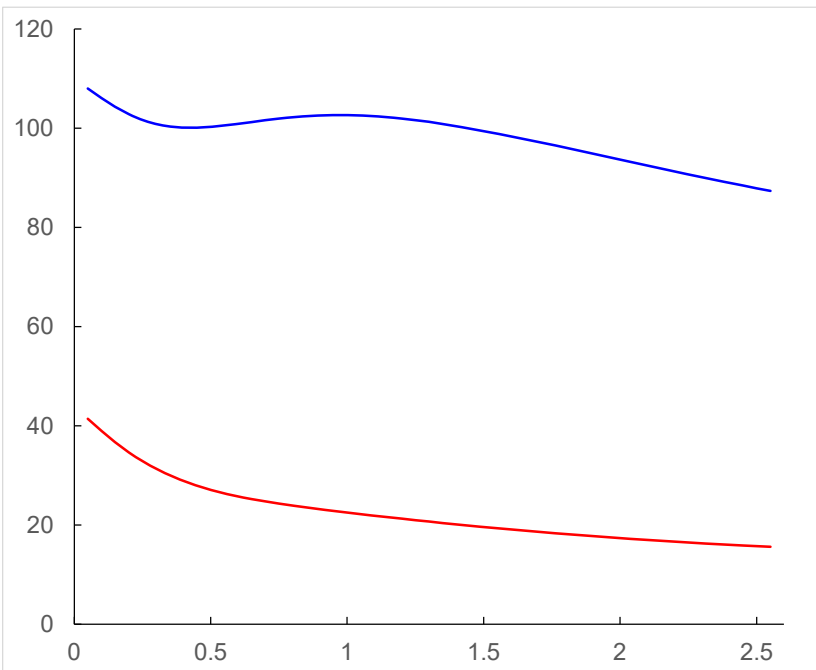

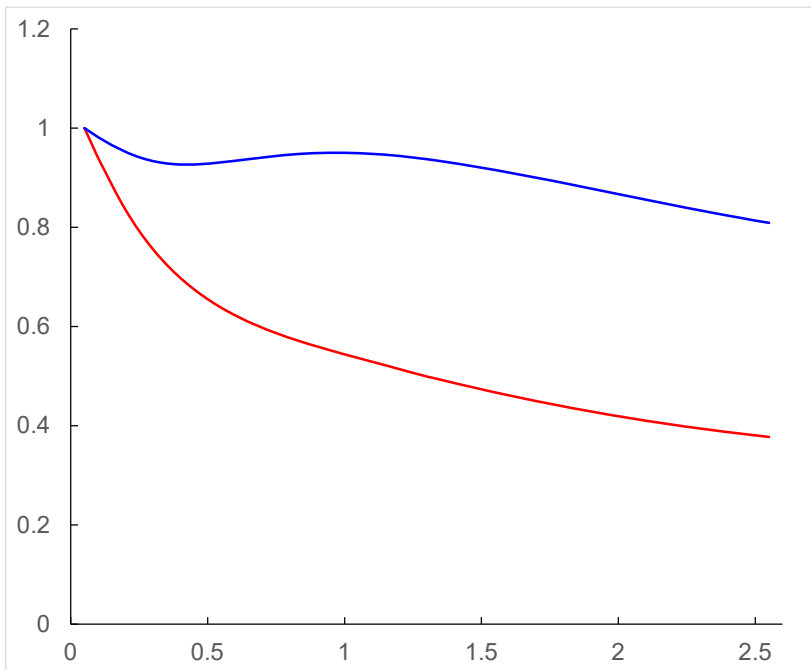

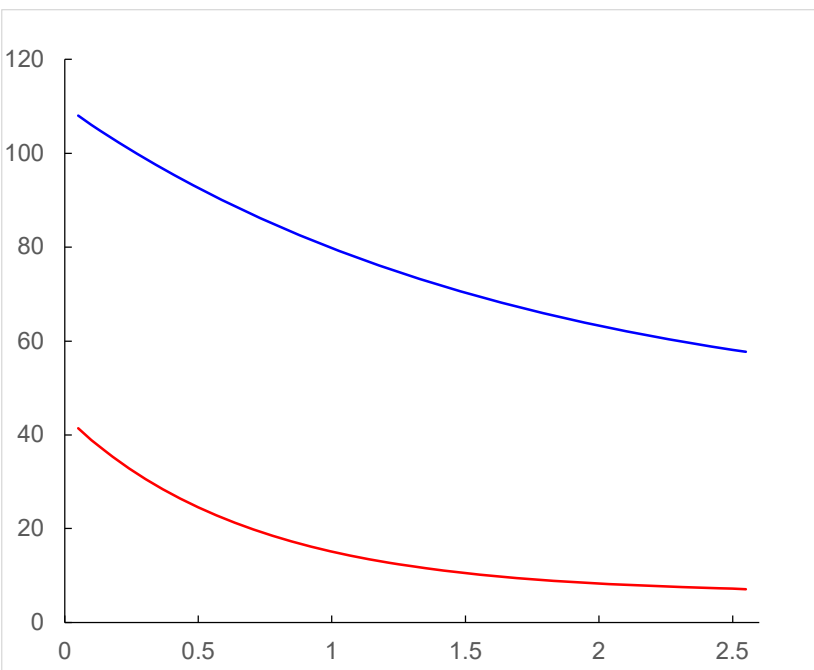

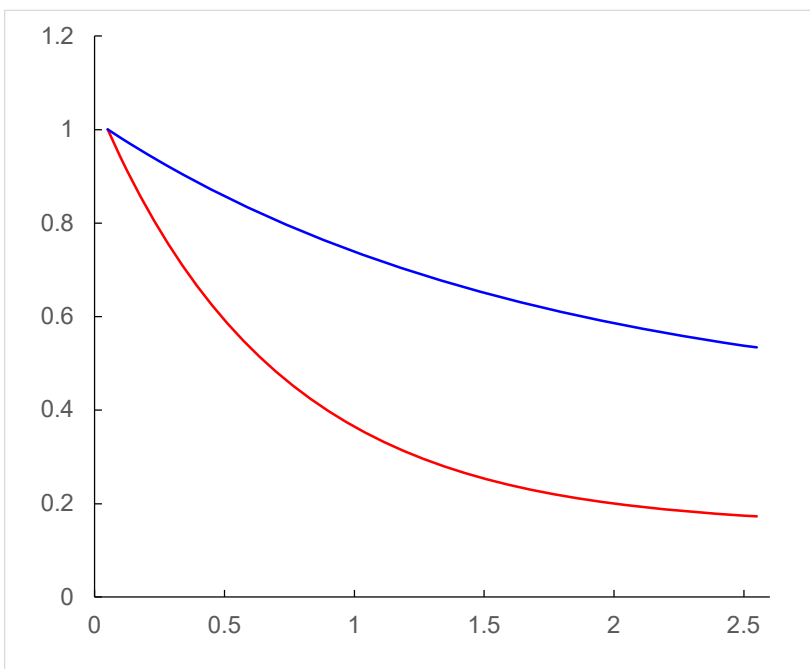

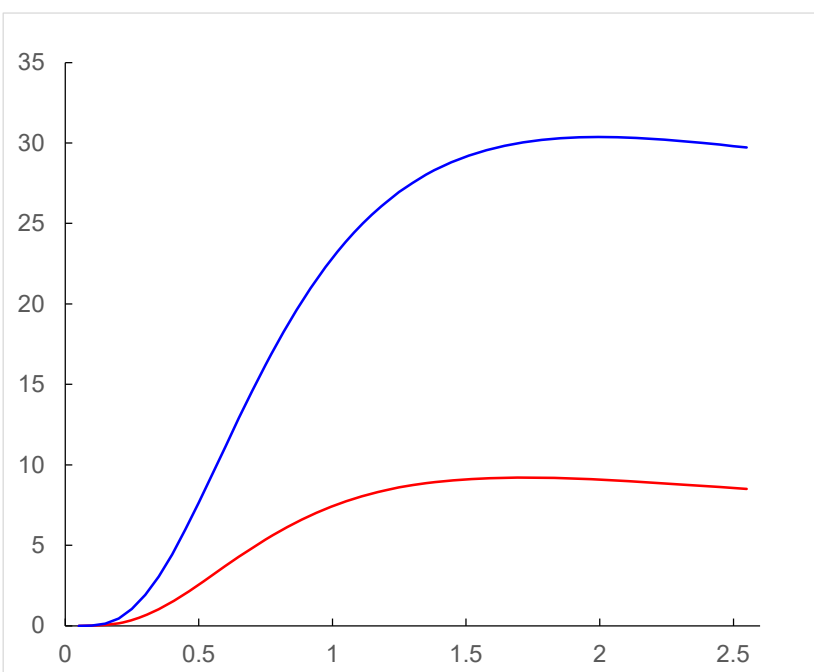

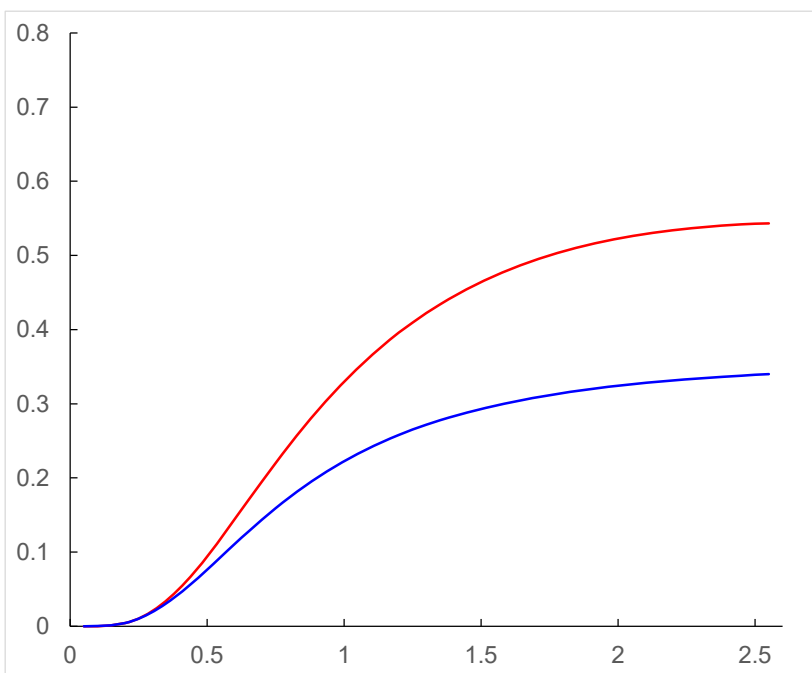

t	FRP_1	FRP_2	SRP_1	SRP_2	RRP_1	RRP_2	rel_RRP_1	rel_RRP_2	rel_FRP_1	rel_FRP_2
0	0	0	0	0	0	0	0	0	0	0
0.05	15.6678	71.5706	20.8904	52.2259	36.5582	123.7965	0.010445	0.011254	0.010445	0.011928
0.1	31.1719	142.2874	41.5625	103.9063	72.7344	246.1937	0.020781	0.022381	0.020781	0.023715
0.15	46.514	212.1607	62.0187	155.0468	108.5327	367.2075	0.031009	0.033383	0.031009	0.03536
0.2	61.696	281.2006	82.2613	205.6532	143.9573	486.8538	0.041131	0.044259	0.041131	0.046867
0.25	76.7193	349.4169	102.2924	255.731	179.0117	605.1479	0.051146	0.055013	0.051146	0.058236
0.3	91.5857	416.8194	122.1143	305.2857	213.7	722.1051	0.061057	0.065646	0.061057	0.06947
0.35	106.2969	483.418	141.7291	354.3229	248.026	837.7409	0.070865	0.076158	0.070865	0.08057
0.4	120.8543	549.2222	161.1391	402.8478	281.9934	952.07	0.08057	0.086552	0.08057	0.091537
0.45	135.2598	614.2414	180.3463	450.8658	315.6061	1065.107	0.090173	0.096828	0.090173	0.102374
0.5	149.5147	678.485	199.3529	498.3824	348.8676	1176.867	0.099676	0.106988	0.099676	0.113081
0.55	163.6208	741.9624	218.161	545.4025	381.7818	1287.365	0.109081	0.117033	0.109081	0.12366
0.6	177.5795	804.6825	236.7726	591.9316	414.3521	1396.614	0.118386	0.126965	0.118386	0.134114
0.65	191.3924	866.6545	255.1899	637.9747	446.5823	1504.629	0.127595	0.136784	0.127595	0.144442
0.7	205.061	927.8872	273.4147	683.5368	478.4757	1611.424	0.136707	0.146493	0.136707	0.154648
0.75	218.5869	988.3896	291.4492	728.623	510.0361	1717.013	0.145725	0.156092	0.145725	0.164732
0.8	231.9715	1048.17	309.2953	773.2383	541.2668	1821.409	0.154648	0.165583	0.154648	0.174695
0.85	245.2163	1107.238	326.955	817.3876	572.1713	1924.625	0.163478	0.174966	0.163478	0.18454
0.9	258.3227	1165.601	344.4303	861.0757	602.753	2026.676	0.172215	0.184243	0.172215	0.194267
0.95	271.2922	1223.268	361.723	904.3075	633.0152	2127.575	0.180861	0.193416	0.180861	0.203878
1	284.1263	1280.246	378.8351	947.0877	662.9614	2227.334	0.189418	0.202485	0.189418	0.213374
1.05	296.8263	1336.546	395.7684	989.4211	692.5947	2325.967	0.197884	0.211452	0.197884	0.222758
1.1	309.3937	1392.173	412.5249	1031.312	721.9186	2423.486	0.206262	0.220317	0.206262	0.232029
1.15	321.8298	1447.138	429.1064	1072.766	750.9362	2519.903	0.214553	0.229082	0.214553	0.24119
1.2	334.136	1501.446	445.5146	1113.787	779.6506	2615.233	0.222757	0.237748	0.222757	0.250241
1.25	346.3136	1555.107	461.7515	1154.379	808.0651	2709.486	0.230876	0.246317	0.230876	0.259184
1.3	358.3641	1608.127	477.8188	1194.547	836.1829	2802.674	0.238909	0.254789	0.238909	0.268021
1.35	370.2887	1660.515	493.7182	1234.296	864.0069	2894.811	0.246859	0.263165	0.246859	0.276753
1.4	382.0887	1712.279	509.4516	1273.629	891.5403	2985.908	0.254726	0.271446	0.254726	0.28538
1.45	393.7655	1763.424	525.0207	1312.552	918.7862	3075.976	0.26251	0.279634	0.26251	0.293904
1.5	405.3203	1813.96	540.4271	1351.068	945.7474	3165.028	0.270214	0.28773	0.270214	0.302327
1.55	416.7544	1863.893	555.6726	1389.181	972.427	3253.074	0.277836	0.295734	0.277836	0.310649
1.6	428.0691	1913.23	570.7588	1426.897	998.8279	3340.127	0.285379	0.303648	0.285379	0.318872
1.65	439.2656	1961.979	585.6875	1464.219	1024.953	3426.198	0.292844	0.311473	0.292844	0.326997
1.7	450.3452	2010.146	600.4603	1501.151	1050.806	3511.297	0.30023	0.319209	0.30023	0.335024
1.75	461.309	2057.739	615.0787	1537.697	1076.388	3595.436	0.307539	0.326858	0.307539	0.342957
1.8	472.1583	2104.764	629.5444	1573.861	1101.703	3678.625	0.314772	0.33442	0.314772	0.350794
1.85	482.8943	2151.228	643.8591	1609.648	1126.753	3760.876	0.32193	0.341898	0.32193	0.358538
1.9	493.5182	2197.138	658.0242	1645.061	1151.542	3842.198	0.329012	0.349291	0.329012	0.36619
1.95	504.031	2242.5	672.0414	1680.104	1176.072	3922.604	0.336021	0.3566	0.336021	0.37375
2	514.4341	2287.321	685.9122	1714.78	1200.346	4002.101	0.342956	0.363827	0.342956	0.38122
2.05	524.7285	2331.608	699.638	1749.095	1224.367	4080.703	0.349819	0.370973	0.349819	0.388601
2.1	534.9154	2375.366	713.2205	1783.051	1248.136	4158.417	0.35661	0.378038	0.35661	0.395894
2.15	544.9959	2418.602	726.6612	1816.653	1271.657	4235.255	0.363331	0.385023	0.363331	0.4031
2.2	554.9711	2461.322	739.9614	1849.904	1294.933	4311.226	0.369981	0.39193	0.369981	0.41022
2.25	564.8421	2503.533	753.1228	1882.807	1317.965	4386.34	0.376561	0.398758	0.376561	0.417256
2.3	574.61	2545.241	766.1466	1915.367	1340.757	4460.607	0.383073	0.40551	0.383073	0.424207
2.35	584.2758	2586.45	779.0344	1947.586	1363.31	4534.037	0.389517	0.412185	0.389517	0.431075
2.4	593.8407	2627.169	791.7876	1979.469	1385.628	4606.638	0.395894	0.418785	0.395894	0.437861
2.45	603.3057	2667.401	804.4076	2011.019	1407.713	4678.42	0.402204	0.425311	0.402204	0.444567
2.5	612.6718	2707.154	816.8958	2042.24	1429.568	4749.393	0.408448	0.431763	0.408448	0.451192
2.55	621.9401	2746.432	829.2535	2073.134	1451.194	4819.566	0.414627	0.438142	0.414627	0.457739
2.6	631.1116	2785.242	841.4822	2103.705	1472.594	4888.948	0.420741	0.44445	0.420741	0.464207
2.65	640.1873	2823.589	853.5831	2133.958	1493.77	4957.547	0.426792	0.450686	0.426792	0.470598
2.7	649.1682	2861.479	865.5576	2163.894	1514.726	5025.373	0.432779	0.456852	0.432779	0.476913
2.75	658.0553	2898.917	877.4071	2193.518	1535.462	5092.434	0.438704	0.462949	0.438704	0.483153
2.8	666.8496	2935.908	889.1328	2222.832	1555.982	5158.74	0.444566	0.468976	0.444566	0.489318
2.85	675.552	2972.457	900.736	2251.84	1576.288	5224.297	0.450368	0.474936	0.450368	0.49541
2.9	684.1635	3008.571	912.218	2280.545	1596.382	5289.116	0.456109	0.480829	0.456109	0.501429
2.95	692.685	3044.254	923.58	2308.95	1616.265	5353.204	0.46179	0.486655	0.46179	0.507376
3	701.1176	3079.512	934.8234	2337.059	1635.941	5416.57	0.467412	0.492415	0.467412	0.513252
3.05	709.4621	3114.349	945.9494	2364.874	1655.412	5479.222	0.472975	0.498111	0.472975	0.519058
3.1	717.7194	3148.77	956.9591	2392.398	1674.679	5541.168	0.47848	0.503743	0.47848	0.524795
3.15	725.8904	3182.781	967.8539	2419.635	1693.744	5602.415	0.483927	0.50931	0.483927	0.530463
3.2	733.9761	3216.386	978.6348	2446.587	1712.611	5662.973	0.489317	0.514816	0.489317	0.536064
3.25	741.9774	3249.59	989.3032	2473.258	1731.281	5722.848	0.494652	0.520259	0.494652	0.541598
3.3	749.8951	3282.398	999.8601	2499.65	1749.755	5782.048	0.49993	0.525641	0.49993	0.547066
3.35	757.73	3314.815	1010.307	2525.767	1768.037	5840.581	0.505153	0.530962	0.505153	0.552469
3.4	765.4832	3346.845	1020.644	2551.611	1786.127	5898.455	0.510322	0.536223	0.510322	0.557807
3.45	773.1553	3378.493	1030.874	2577.185	1804.029	5955.677	0.515437	0.541425	0.515437	0.563082
3.5	780.7474	3409.763	1040.997	2602.491	1821.744	6012.254	0.520498	0.546569	0.520498	0.568294
3.55	788.2601	3440.66	1051.013	2627.534	1839.274	6068.194	0.525507	0.551654	0.525507	0.573443
3.6	795.6943	3471.189	1060.926	2652.314	1856.62	6123.504	0.530463	0.556682	0.530463	0.578532
3.65	803.0509	3501.354	1070.735	2676.836	1873.786	6178.191	0.535367	0.561654	0.535367	0.583559
3.7	810.3307	3531.159	1080.441	2701.102	1890.772	6232.261	0.54022	0.566569	0.54022	0.588527
3.75	817.5344	3560.608	1090.046	2725.115	1907.58	6285.723	0.545023	0.571429	0.545023	0.593435
3.8	824.6629	3589.707	1099.551	2748.876	1924.213	6338.583	0.549775	0.576235	0.549775	0.598284
3.85	831.7169	3618.458	1108.956	2772.39	1940.673	6390.847	0.554478	0.580986	0.554478	0.603076
3.9	838.6972	3646.866	1118.263	2795.657	1956.96	6442.523	0.559131	0.585684	0.559131	0.607811
3.95	845.6047	3674.935	1127.473	2818.682	1973.078	6493.617	0.563736	0.590329	0.563736	0.612489
4	852.4399	3702.669	1136.587	2841.466	1989.027	6544.136	0.568293	0.594921	0.568293	0.617112
4.05	859.2038	3730.073	1145.605	2864.013	2004.809	6594.085	0.572803	0.599462	0.572803	0.621679
4.1	865.897	3757.149	1154.529	2886.324	2020.426	6643.473	0.577265	0.603952	0.577265	0.626192
4.15	872.5204	3783.903	1163.361	2908.401	2035.881	6692.304	0.58168	0.608391	0.58168	0.630651
4.2	879.0745	3810.338	1172.099	2930.248	2051.174	6740.586	0.58605	0.612781	0.58605	0.635056
4.25	885.5602	3836.457	1180.747	2951.867	2066.307	6788.324	0.590373	0.61712	0.590373	0.639409

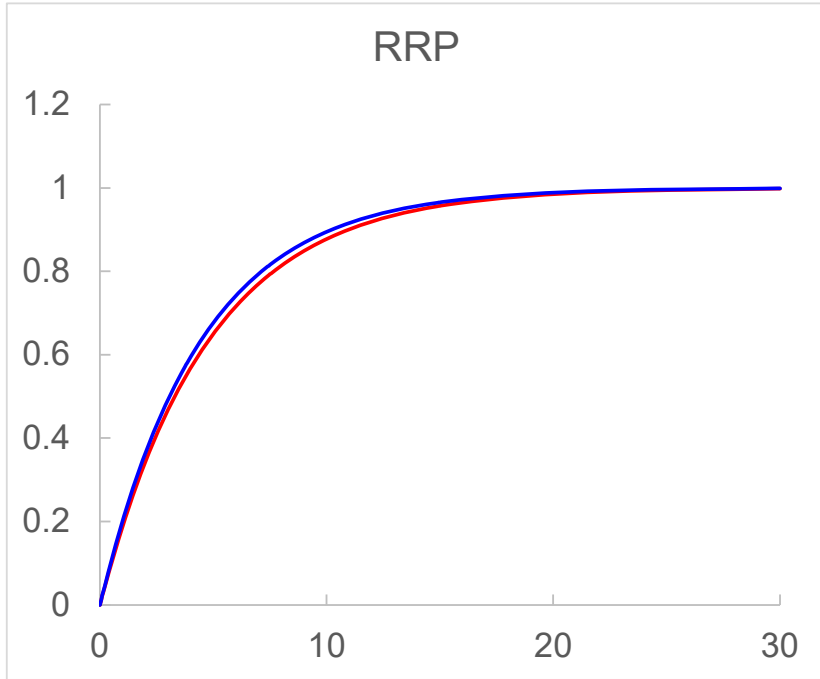

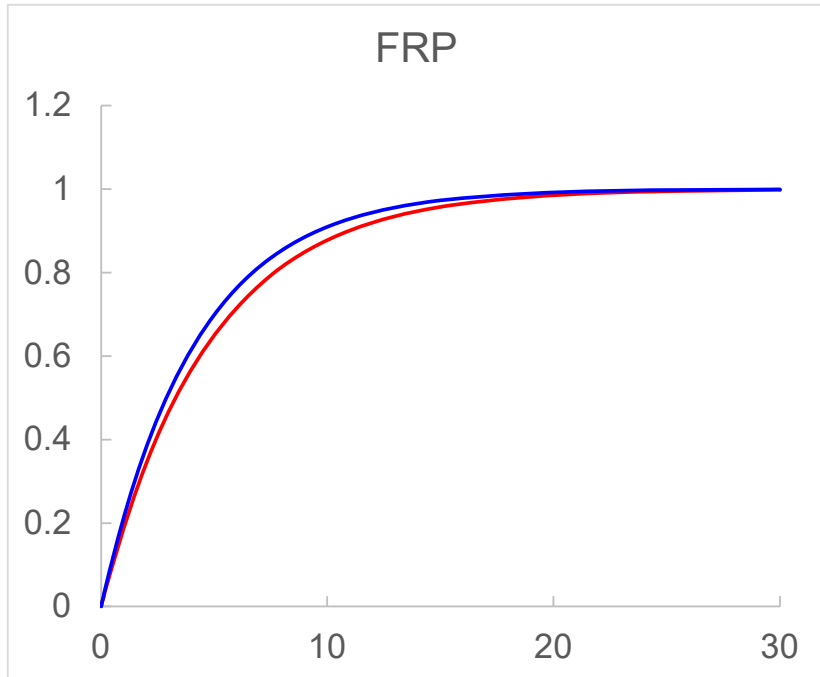

4.3	891.9781	3862.265	1189.304	2973.26	2081.282	6835.525	0.594652	0.621411	0.594652	0.643711
4.35	898.329	3887.764	1197.772	2994.43	2096.101	6882.194	0.598886	0.625654	0.598886	0.647961
4.4	904.6136	3912.96	1206.151	3015.379	2110.765	6928.339	0.603076	0.629849	0.603076	0.65216
4.45	910.8325	3937.855	1214.443	3036.108	2125.276	6973.963	0.607222	0.633997	0.607222	0.656309
4.5	916.9864	3962.453	1222.649	3056.621	2139.635	7019.075	0.611324	0.638098	0.611324	0.660409
4.55	923.0761	3986.758	1230.768	3076.92	2153.844	7063.678	0.615384	0.642153	0.615384	0.66446
4.6	929.1022	4010.773	1238.803	3097.007	2167.905	7107.78	0.619401	0.646162	0.619401	0.668462
4.65	935.0653	4034.501	1246.754	3116.884	2181.819	7151.386	0.623377	0.650126	0.623377	0.672417
4.7	940.9662	4057.947	1254.622	3136.554	2195.588	7194.5	0.627311	0.654045	0.627311	0.676324
4.75	946.8054	4081.112	1262.407	3156.018	2209.213	7237.13	0.631204	0.657921	0.631204	0.680185
4.8	952.5836	4104.001	1270.111	3175.279	2222.695	7279.28	0.635056	0.661753	0.635056	0.684
4.85	958.3014	4126.618	1277.735	3194.338	2236.037	7320.956	0.638868	0.665541	0.638868	0.68777
4.9	963.9596	4148.964	1285.279	3213.199	2249.239	7362.163	0.64264	0.669288	0.64264	0.691494
4.95	969.5586	4171.044	1292.745	3231.862	2262.303	7402.906	0.646372	0.672991	0.646372	0.695174
5	975.0992	4192.861	1300.132	3250.331	2275.231	7443.191	0.650066	0.676654	0.650066	0.69881
5.05	980.5818	4214.417	1307.443	3268.606	2288.024	7483.023	0.653721	0.680275	0.653721	0.702403
5.1	986.0073	4235.716	1314.676	3286.691	2300.684	7522.407	0.657338	0.683855	0.657338	0.705953
5.15	991.376	4256.761	1321.835	3304.587	2313.211	7561.348	0.660917	0.687395	0.660917	0.70946
5.2	996.6887	4277.556	1328.918	3322.296	2325.607	7599.851	0.664459	0.690896	0.664459	0.712926
5.25	1001.946	4298.102	1335.928	3339.82	2337.874	7637.921	0.667964	0.694356	0.667964	0.71635
5.3	1007.148	4318.403	1342.864	3357.16	2350.012	7675.563	0.671432	0.697778	0.671432	0.719734
5.35	1012.296	4338.461	1349.728	3374.32	2362.024	7712.782	0.674864	0.701162	0.674864	0.723077
5.4	1017.39	4358.281	1356.52	3391.301	2373.911	7749.582	0.67826	0.704507	0.67826	0.72638
5.45	1022.431	4377.864	1363.242	3408.104	2385.673	7785.968	0.681621	0.707815	0.681621	0.729644
5.5	1027.419	4397.214	1369.893	3424.731	2397.312	7821.945	0.684946	0.711086	0.684946	0.732869
5.55	1032.356	4416.332	1376.474	3441.185	2408.83	7857.518	0.688237	0.71432	0.688237	0.736055
5.6	1037.24	4435.223	1382.987	3457.468	2420.227	7892.691	0.691493	0.717517	0.691493	0.739204
5.65	1042.074	4453.888	1389.432	3473.58	2431.506	7927.468	0.694716	0.720679	0.694716	0.742315
5.7	1046.857	4472.331	1395.809	3489.523	2442.666	7961.854	0.697905	0.723805	0.697905	0.745389
5.75	1051.59	4490.554	1402.12	3505.3	2453.71	7995.854	0.70106	0.726896	0.70106	0.748426
5.8	1056.274	4508.559	1408.365	3520.913	2464.639	8029.472	0.704183	0.729952	0.704183	0.751427
5.85	1060.909	4526.35	1414.545	3536.362	2475.454	8062.712	0.707272	0.732974	0.707272	0.754392
5.9	1065.495	4543.928	1420.66	3551.65	2486.155	8095.578	0.71033	0.735962	0.71033	0.757321
5.95	1070.034	4561.297	1426.711	3566.778	2496.745	8128.075	0.713356	0.738916	0.713356	0.760216
6	1074.525	4578.458	1432.7	3581.749	2507.224	8160.207	0.71635	0.741837	0.71635	0.763076
6.05	1078.969	4595.415	1438.625	3596.563	2517.594	8191.977	0.719313	0.744725	0.719313	0.765902
6.1	1083.367	4612.169	1444.489	3611.222	2527.855	8223.391	0.722244	0.747581	0.722244	0.768695
6.15	1087.718	4628.724	1450.291	3625.728	2538.009	8254.452	0.725146	0.750405	0.725146	0.771454
6.2	1092.025	4645.081	1456.033	3640.082	2548.058	8285.163	0.728016	0.753197	0.728016	0.77418
6.25	1096.286	4661.243	1461.715	3654.287	2558.001	8315.53	0.730857	0.755957	0.730857	0.776874
6.3	1100.503	4677.212	1467.337	3668.343	2567.84	8345.555	0.733669	0.758687	0.733669	0.779535
6.35	1104.676	4692.991	1472.901	3682.252	2577.577	8375.244	0.73645	0.761386	0.73645	0.782165
6.4	1108.805	4708.582	1478.407	3696.017	2587.212	8404.598	0.739203	0.764054	0.739203	0.784764
6.45	1112.891	4723.986	1483.855	3709.637	2596.746	8433.623	0.741927	0.766693	0.741927	0.787331
6.5	1116.935	4739.207	1489.246	3723.115	2606.181	8462.322	0.744623	0.769302	0.744623	0.789868
6.55	1120.936	4754.247	1494.581	3736.452	2615.517	8490.699	0.74729	0.771882	0.74729	0.792374
6.6	1124.895	4769.106	1499.86	3749.65	2624.755	8518.757	0.74993	0.774432	0.74993	0.794851
6.65	1128.813	4783.789	1505.084	3762.71	2633.897	8546.499	0.752542	0.776954	0.752542	0.797298
6.7	1132.69	4798.297	1510.254	3775.634	2642.944	8573.931	0.755127	0.779448	0.755127	0.799716
6.75	1136.527	4812.631	1515.369	3788.423	2651.896	8601.054	0.757685	0.781914	0.757685	0.802105
6.8	1140.323	4826.794	1520.431	3801.078	2660.755	8627.872	0.760216	0.784352	0.760216	0.804466
6.85	1144.08	4840.789	1525.44	3813.601	2669.521	8654.39	0.76272	0.786763	0.76272	0.806798
6.9	1147.798	4854.616	1530.397	3825.993	2678.195	8680.609	0.765199	0.789146	0.765199	0.809103
6.95	1151.477	4868.279	1535.302	3838.256	2686.779	8706.535	0.767651	0.791503	0.767651	0.81138
7	1155.117	4881.779	1540.156	3850.39	2695.273	8732.169	0.770078	0.793834	0.770078	0.81363
7.05	1158.72	4895.117	1544.959	3862.398	2703.679	8757.516	0.77248	0.796138	0.77248	0.815853
7.1	1162.284	4908.297	1549.712	3874.281	2711.997	8782.577	0.774856	0.798416	0.774856	0.818049
7.15	1165.812	4921.319	1554.416	3886.039	2720.227	8807.358	0.777208	0.800669	0.777208	0.82022
7.2	1169.302	4934.186	1559.07	3897.675	2728.372	8831.861	0.779535	0.802896	0.779535	0.822364
7.25	1172.757	4946.9	1563.675	3909.189	2736.432	8856.088	0.781838	0.805099	0.781838	0.824483
7.3	1176.175	4959.461	1568.233	3920.582	2744.408	8880.044	0.784116	0.807277	0.784116	0.826577
7.35	1179.557	4971.873	1572.743	3931.857	2752.3	8903.73	0.786371	0.80943	0.786371	0.828646
7.4	1182.904	4984.137	1577.206	3943.014	2760.11	8927.151	0.788603	0.811559	0.788603	0.83069
7.45	1186.216	4996.255	1581.622	3954.054	2767.838	8950.309	0.790811	0.813664	0.790811	0.832709
7.5	1189.494	5008.228	1585.992	3964.979	2775.486	8973.208	0.792996	0.815746	0.792996	0.834705
7.55	1192.737	5020.058	1590.316	3975.79	2783.053	8995.849	0.795158	0.817804	0.795158	0.836676
7.6	1195.947	5031.748	1594.595	3986.488	2790.542	9018.236	0.797298	0.81984	0.797298	0.838625
7.65	1199.122	5043.297	1598.83	3997.075	2797.952	9040.372	0.799415	0.821852	0.799415	0.84055
7.7	1202.265	5054.709	1603.02	4007.55	2805.285	9062.26	0.80151	0.823842	0.80151	0.842452
7.75	1205.375	5065.985	1607.167	4017.917	2812.542	9083.902	0.803583	0.825809	0.803583	0.844331
7.8	1208.452	5077.126	1611.27	4028.175	2819.722	9105.301	0.805635	0.827755	0.805635	0.846188
7.85	1211.498	5088.135	1615.33	4038.326	2826.828	9126.46	0.807665	0.829678	0.807665	0.848022
7.9	1214.511	5099.012	1619.348	4048.371	2833.859	9147.382	0.809674	0.83158	0.809674	0.849835
7.95	1217.493	5109.759	1623.324	4058.31	2840.817	9168.07	0.811662	0.833461	0.811662	0.851627
8	1220.444	5120.379	1627.259	4068.147	2847.703	9188.525	0.813629	0.83532	0.813629	0.853396
8.05	1223.364	5130.871	1631.152	4077.88	2854.516	9208.751	0.815576	0.837159	0.815576	0.855145
8.1	1226.254	5141.238	1635.005	4087.512	2861.258	9228.75	0.817502	0.838977	0.817502	0.856873
8.15	1229.113	5151.482	1638.817	4097.043	2867.93	9248.525	0.819409	0.840775	0.819409	0.85858
8.2	1231.942	5161.604	1642.59	4106.474	2874.532	9268.078	0.821295	0.842553	0.821295	0.860267
8.25	1234.742	5171.604	1646.323	4115.807	2881.065	9287.412	0.823161	0.84431	0.823161	0.861934
8.3	1237.513	5181.486	1650.017	4125.043	2887.53	9306.529	0.825009	0.846048	0.825009	0.863581
8.35	1240.255	5191.249	1653.673	4134.182	2893.927	9325.431	0.826836	0.847766	0.826836	0.865208
8.4	1242.968	5200.896	1657.29	4143.226	2900.258	9344.122	0.828645	0.849466	0.828645	0.866816
8.45	1245.652	5210.429	1660.87	4152.175	2906.522	9362.603	0.830435	0.851146	0.830435	0.868405
8.5	1248.309	5219.847	1664.412	4161.03	2912.721	9380.877	0.832206	0.852807	0.832206	0.869974
8.55	1250.938	5229.153	1667.917	4169.794	2918.856	9398.946	0.833959	0.85445	0.833959	0.871525
8.6	1253.54	5238.348	1671.386	4178.465	2924.926	9416.813	0.835693	0.856074	0.835693	0.873058

8.65	1256.114	5247.433	1674.819	4187.046	2930.932	9434.479	0.837409	0.85768	0.837409	0.874572
8.7	1258.661	5256.41	1678.215	4195.538	2936.876	9451.948	0.839108	0.859268	0.839108	0.876068
8.75	1261.182	5265.28	1681.576	4203.941	2942.758	9469.22	0.840788	0.860838	0.840788	0.877547
8.8	1263.677	5274.044	1684.902	4212.256	2948.579	9486.3	0.842451	0.862391	0.842451	0.879007
8.85	1266.145	5282.704	1688.194	4220.484	2954.339	9503.187	0.844097	0.863926	0.844097	0.880451
8.9	1268.588	5291.26	1691.45	4228.626	2960.038	9519.886	0.845725	0.865444	0.845725	0.881877
8.95	1271.005	5299.714	1694.673	4236.683	2965.678	9536.397	0.847337	0.866945	0.847337	0.883286
9	1273.397	5308.067	1697.862	4244.656	2971.259	9552.723	0.848931	0.868429	0.848931	0.884678
9.05	1275.764	5316.321	1701.018	4252.546	2976.782	9568.867	0.850509	0.869897	0.850509	0.886053
9.1	1278.106	5324.476	1704.141	4260.353	2982.247	9584.829	0.852071	0.871348	0.852071	0.887413
9.15	1280.424	5332.534	1707.232	4268.079	2987.655	9600.613	0.853616	0.872783	0.853616	0.888756
9.2	1282.717	5340.496	1710.29	4275.724	2993.007	9616.22	0.855145	0.874202	0.855145	0.890083
9.25	1284.987	5348.363	1713.316	4283.289	2998.302	9631.652	0.856658	0.875605	0.856658	0.891394
9.3	1287.233	5356.136	1716.31	4290.775	3003.543	9646.911	0.858155	0.876992	0.858155	0.892689
9.35	1289.455	5363.816	1719.273	4298.183	3008.728	9661.999	0.859637	0.878364	0.859637	0.893969
9.4	1291.654	5371.405	1722.206	4305.514	3013.86	9676.918	0.861103	0.87972	0.861103	0.895234
9.45	1293.83	5378.903	1725.107	4312.768	3018.937	9691.671	0.862554	0.881061	0.862554	0.896484
9.5	1295.984	5386.312	1727.978	4319.946	3023.962	9706.258	0.863989	0.882387	0.863989	0.897719
9.55	1298.115	5393.632	1730.82	4327.049	3028.935	9720.681	0.86541	0.883698	0.86541	0.898939
9.6	1300.224	5400.865	1733.631	4334.078	3033.855	9734.943	0.866816	0.884995	0.866816	0.900144
9.65	1302.31	5408.012	1736.414	4341.034	3038.724	9749.046	0.868207	0.886277	0.868207	0.901335
9.7	1304.375	5415.073	1739.167	4347.917	3043.542	9762.99	0.869583	0.887545	0.869583	0.902512
9.75	1306.418	5422.05	1741.891	4354.728	3048.31	9776.779	0.870946	0.888798	0.870946	0.903675
9.8	1308.44	5428.944	1744.587	4361.468	3053.028	9790.413	0.872294	0.890038	0.872294	0.904824
9.85	1310.441	5435.756	1747.255	4368.138	3057.696	9803.894	0.873628	0.891263	0.873628	0.905959
9.9	1312.421	5442.487	1749.895	4374.738	3062.316	9817.224	0.874948	0.892475	0.874948	0.907081
9.95	1314.381	5449.137	1752.507	4381.269	3066.888	9830.406	0.876254	0.893673	0.876254	0.90819
10	1316.319	5455.708	1755.093	4387.731	3071.412	9843.439	0.877546	0.894858	0.877546	0.909285
10.05	1318.238	5462.201	1757.651	4394.127	3075.889	9856.327	0.878825	0.89603	0.878825	0.910367
10.1	1320.137	5468.616	1760.182	4400.455	3080.319	9869.071	0.880091	0.897188	0.880091	0.911436
10.15	1322.015	5474.954	1762.687	4406.717	3084.702	9881.672	0.881343	0.898334	0.881343	0.912492
10.2	1323.874	5481.217	1765.166	4412.914	3089.04	9894.132	0.882583	0.899467	0.882583	0.913536
10.25	1325.714	5487.405	1767.619	4419.047	3093.333	9906.452	0.883809	0.900587	0.883809	0.914568
10.3	1327.534	5493.52	1770.046	4425.115	3097.58	9918.635	0.885023	0.901694	0.885023	0.915587
10.35	1329.336	5499.561	1772.448	4431.12	3101.784	9930.681	0.886224	0.902789	0.886224	0.916594
10.4	1331.119	5505.531	1774.825	4437.062	3105.943	9942.592	0.887412	0.903872	0.887412	0.917588
10.45	1332.883	5511.429	1777.177	4442.942	3110.059	9954.371	0.888588	0.904943	0.888588	0.918572
10.5	1334.628	5517.257	1779.504	4448.76	3114.132	9966.017	0.889752	0.906002	0.889752	0.919543
10.55	1336.355	5523.015	1781.807	4454.518	3118.163	9977.533	0.890904	0.907048	0.890904	0.920503
10.6	1338.065	5528.705	1784.086	4460.216	3122.151	9988.921	0.892043	0.908084	0.892043	0.921451
10.65	1339.756	5534.327	1786.342	4465.854	3126.098	10000.18	0.893171	0.909107	0.893171	0.922388
10.7	1341.43	5539.882	1788.573	4471.433	3130.003	10011.31	0.894287	0.91012	0.894287	0.923314
10.75	1343.086	5545.37	1790.782	4476.954	3133.868	10022.32	0.895391	0.91112	0.895391	0.924228
10.8	1344.725	5550.793	1792.967	4482.417	3137.692	10033.21	0.896483	0.91211	0.896483	0.925132
10.85	1346.347	5556.151	1795.129	4487.824	3141.477	10043.97	0.897565	0.913089	0.897565	0.926025
10.9	1347.952	5561.446	1797.269	4493.173	3145.221	10054.62	0.898635	0.914056	0.898635	0.926908
10.95	1349.54	5566.677	1799.387	4498.467	3148.927	10065.14	0.899693	0.915013	0.899693	0.92778
11	1351.112	5571.846	1801.482	4503.706	3152.594	10075.55	0.900741	0.915959	0.900741	0.928641
11.05	1352.667	5576.953	1803.556	4508.89	3156.223	10085.84	0.901778	0.916895	0.901778	0.929492
11.1	1354.206	5581.999	1805.608	4514.019	3159.814	10096.02	0.902804	0.91782	0.902804	0.930333
11.15	1355.729	5586.986	1807.638	4519.096	3163.367	10106.08	0.903819	0.918735	0.903819	0.931164
11.2	1357.236	5591.912	1809.648	4524.119	3166.883	10116.03	0.904824	0.919639	0.904824	0.931985
11.25	1358.727	5596.78	1811.636	4529.089	3170.363	10125.87	0.905818	0.920534	0.905818	0.932797
11.3	1360.202	5601.59	1813.603	4534.008	3173.806	10135.6	0.906802	0.921418	0.906802	0.933598
11.35	1361.663	5606.342	1815.55	4538.876	3177.213	10145.22	0.907775	0.922293	0.907775	0.93439
11.4	1363.108	5611.038	1817.477	4543.692	3180.584	10154.73	0.908738	0.923157	0.908738	0.935173
11.45	1364.538	5615.678	1819.383	4548.458	3183.921	10164.14	0.909692	0.924012	0.909692	0.935946
11.5	1365.952	5620.262	1821.27	4553.175	3187.222	10173.44	0.910635	0.924858	0.910635	0.93671
11.55	1367.353	5624.792	1823.137	4557.842	3190.489	10182.63	0.911568	0.925694	0.911568	0.937465
11.6	1368.738	5629.267	1824.984	4562.46	3193.722	10191.73	0.912492	0.926521	0.912492	0.938211
11.65	1370.109	5633.69	1826.812	4567.03	3196.921	10200.72	0.913406	0.927338	0.913406	0.938948
11.7	1371.466	5638.059	1828.621	4571.553	3200.087	10209.61	0.914311	0.928147	0.914311	0.939677
11.75	1372.808	5642.376	1830.411	4576.028	3203.22	10218.4	0.915206	0.928946	0.915206	0.940396
11.8	1374.137	5646.642	1832.183	4580.457	3206.32	10227.1	0.916091	0.929736	0.916091	0.941107
11.85	1375.452	5650.857	1833.936	4584.839	3209.387	10235.7	0.916968	0.930518	0.916968	0.94181
11.9	1376.753	5655.022	1835.67	4589.175	3212.423	10244.2	0.917835	0.931291	0.917835	0.942504
11.95	1378.04	5659.137	1837.387	4593.466	3215.426	10252.6	0.918693	0.932055	0.918693	0.94319
12	1379.314	5663.203	1839.085	4597.713	3218.399	10260.92	0.919543	0.932811	0.919543	0.943867
12.05	1380.574	5667.221	1840.766	4601.915	3221.34	10269.14	0.920383	0.933558	0.920383	0.944537
12.1	1381.822	5671.19	1842.429	4606.073	3224.251	10277.26	0.921215	0.934297	0.921215	0.945198
12.15	1383.056	5675.112	1844.075	4610.187	3227.131	10285.3	0.922037	0.935027	0.922037	0.945852
12.2	1384.278	5678.988	1845.704	4614.259	3229.981	10293.25	0.922852	0.93575	0.922852	0.946498
12.25	1385.486	5682.817	1847.315	4618.288	3232.802	10301.1	0.923658	0.936464	0.923658	0.947136
12.3	1386.683	5686.6	1848.91	4622.275	3235.593	10308.88	0.924455	0.93717	0.924455	0.947767
12.35	1387.866	5690.339	1850.488	4626.221	3238.354	10316.56	0.925244	0.937869	0.925244	0.94839
12.4	1389.037	5694.033	1852.05	4630.125	3241.087	10324.16	0.926025	0.93856	0.926025	0.949005
12.45	1390.196	5697.682	1853.595	4633.988	3243.792	10331.67	0.926798	0.939243	0.926798	0.949614
12.5	1391.343	5701.288	1855.125	4637.811	3246.468	10339.1	0.927562	0.939918	0.927562	0.950215
12.55	1392.478	5704.852	1856.638	4641.594	3249.116	10346.45	0.928319	0.940586	0.928319	0.950809
12.6	1393.601	5708.372	1858.135	4645.338	3251.737	10353.71	0.929068	0.941246	0.929068	0.951395
12.65	1394.713	5711.851	1859.617	4649.042	3254.33	10360.89	0.929808	0.941899	0.929808	0.951975
12.7	1395.813	5715.288	1861.083	4652.708	3256.896	10368	0.930542	0.942545	0.930542	0.952548
12.75	1396.901	5718.684	1862.534	4656.336	3259.435	10375.02	0.931267	0.943184	0.931267	0.953114
12.8	1397.978	5722.04	1863.97	4659.925	3261.948	10381.97	0.931985	0.943815	0.931985	0.953673
12.85	1399.043	5725.355	1865.391	4663.478	3264.434	10388.83	0.932696	0.944439	0.932696	0.954226
12.9	1400.098	5728.632	1866.797	4666.993	3266.895	10395.62	0.933399	0.945057	0.933399	0.954772
12.95	1401.141	5731.869	1868.188	4670.471	3269.33	10402.34	0.934094	0.945667	0.934094	0.955311

13	1402.174	5735.067	1869.565	4673.913	3271.739	10408.98	0.934783	0.946271	0.934783	0.955844
13.05	1403.196	5738.227	1870.928	4677.319	3274.123	10415.55	0.935464	0.946868	0.935464	0.956371
13.1	1404.207	5741.35	1872.276	4680.689	3276.483	10422.04	0.936138	0.947458	0.936138	0.956892
13.15	1405.207	5744.435	1873.61	4684.025	3278.817	10428.46	0.936805	0.948042	0.936805	0.957406
13.2	1406.198	5747.484	1874.93	4687.325	3281.128	10434.81	0.937465	0.948619	0.937465	0.957914
13.25	1407.177	5750.496	1876.236	4690.591	3283.414	10441.09	0.938118	0.94919	0.938118	0.958416
13.3	1408.147	5753.472	1877.529	4693.823	3285.676	10447.29	0.938765	0.949754	0.938765	0.958912
13.35	1409.106	5756.413	1878.808	4697.021	3287.915	10453.43	0.939404	0.950312	0.939404	0.959402
13.4	1410.056	5759.318	1880.074	4700.186	3290.13	10459.5	0.940037	0.950864	0.940037	0.959886
13.45	1410.995	5762.189	1881.327	4703.317	3292.322	10465.51	0.940663	0.95141	0.940663	0.960365
13.5	1411.925	5765.026	1882.566	4706.416	3294.491	10471.44	0.941283	0.951949	0.941283	0.960838
13.55	1412.845	5767.829	1883.793	4709.483	3296.638	10477.31	0.941897	0.952483	0.941897	0.961305
13.6	1413.755	5770.598	1885.007	4712.517	3298.762	10483.12	0.942503	0.95301	0.942503	0.961766
13.65	1414.656	5773.335	1886.208	4715.52	3300.864	10488.85	0.943104	0.953532	0.943104	0.962222
13.7	1415.547	5776.038	1887.397	4718.491	3302.944	10494.53	0.943698	0.954048	0.943698	0.962673
13.75	1416.43	5778.71	1888.573	4721.432	3305.002	10500.14	0.944286	0.954558	0.944286	0.963118
13.8	1417.303	5781.349	1889.737	4724.342	3307.039	10505.69	0.944868	0.955063	0.944868	0.963558
13.85	1418.166	5783.958	1890.888	4727.221	3309.055	10511.18	0.945444	0.955562	0.945444	0.963993
13.9	1419.021	5786.535	1892.028	4730.07	3311.049	10516.6	0.946014	0.956055	0.946014	0.964422
13.95	1419.867	5789.081	1893.156	4732.89	3313.023	10521.97	0.946578	0.956543	0.946578	0.964847
14	1420.704	5791.597	1894.272	4735.68	3314.976	10527.28	0.947136	0.957025	0.947136	0.965266
14.05	1421.532	5794.083	1895.376	4738.44	3316.908	10532.52	0.947688	0.957502	0.947688	0.96568
14.1	1422.352	5796.539	1896.469	4741.172	3318.821	10537.71	0.948234	0.957974	0.948234	0.96609
14.15	1423.163	5798.966	1897.55	4743.876	3320.713	10542.84	0.948775	0.95844	0.948775	0.966494
14.2	1423.965	5801.364	1898.621	4746.551	3322.586	10547.92	0.94931	0.958901	0.94931	0.966894
14.25	1424.76	5803.733	1899.679	4749.199	3324.439	10552.93	0.94984	0.959357	0.94984	0.967289
14.3	1425.546	5806.075	1900.727	4751.818	3326.273	10557.89	0.950364	0.959808	0.950364	0.967679
14.35	1426.323	5808.388	1901.764	4754.411	3328.087	10562.8	0.950882	0.960254	0.950882	0.968065
14.4	1427.093	5810.674	1902.79	4756.976	3329.883	10567.65	0.951395	0.960695	0.951395	0.968446
14.45	1427.854	5812.932	1903.806	4759.514	3331.66	10572.45	0.951903	0.961131	0.951903	0.968822
14.5	1428.608	5815.163	1904.81	4762.026	3333.418	10577.19	0.952405	0.961563	0.952405	0.969194
14.55	1429.354	5817.368	1905.805	4764.512	3335.158	10581.88	0.952902	0.961989	0.952902	0.969561
14.6	1430.091	5819.547	1906.789	4766.971	3336.88	10586.52	0.953394	0.962411	0.953394	0.969924
14.65	1430.822	5821.699	1907.762	4769.406	3338.584	10591.1	0.953881	0.962828	0.953881	0.970283
14.7	1431.544	5823.826	1908.726	4771.814	3340.27	10595.64	0.954363	0.96324	0.954363	0.970638
14.75	1432.259	5825.927	1909.679	4774.198	3341.938	10600.12	0.95484	0.963648	0.95484	0.970988
14.8	1432.967	5828.004	1910.622	4776.556	3343.589	10604.56	0.955311	0.964051	0.955311	0.971334
14.85	1433.667	5830.056	1911.556	4778.89	3345.223	10608.95	0.955778	0.96445	0.955778	0.971676
14.9	1434.36	5832.083	1912.48	4781.2	3346.84	10613.28	0.95624	0.964844	0.95624	0.972014
14.95	1435.046	5834.086	1913.394	4783.485	3348.44	10617.57	0.956697	0.965234	0.956697	0.972348
15	1435.724	5836.065	1914.299	4785.747	3350.023	10621.81	0.957149	0.965619	0.957149	0.972677
15.05	1436.395	5838.02	1915.194	4787.984	3351.589	10626	0.957597	0.966	0.957597	0.973003
15.1	1437.06	5839.952	1916.08	4790.199	3353.139	10630.15	0.95804	0.966377	0.95804	0.973325
15.15	1437.717	5841.862	1916.956	4792.39	3354.673	10634.25	0.958478	0.96675	0.958478	0.973644
15.2	1438.368	5843.748	1917.824	4794.559	3356.191	10638.31	0.958912	0.967119	0.958912	0.973958
15.25	1439.011	5845.612	1918.682	4796.705	3357.693	10642.32	0.959341	0.967483	0.959341	0.974269
15.3	1439.648	5847.453	1919.531	4798.828	3359.18	10646.28	0.959766	0.967844	0.959766	0.974576
15.35	1440.279	5849.273	1920.372	4800.929	3360.651	10650.2	0.960186	0.9682	0.960186	0.974879
15.4	1440.903	5851.071	1921.204	4803.009	3362.106	10654.08	0.960602	0.968553	0.960602	0.975178
15.45	1441.52	5852.847	1922.027	4805.066	3363.546	10657.91	0.961013	0.968901	0.961013	0.975475
15.5	1442.131	5854.603	1922.841	4807.103	3364.972	10661.71	0.96142	0.969246	0.96142	0.975767
15.55	1442.735	5856.337	1923.647	4809.117	3366.382	10665.45	0.961823	0.969587	0.961823	0.976056
15.6	1443.333	5858.051	1924.445	4811.111	3367.778	10669.16	0.962222	0.969924	0.962222	0.976342
15.65	1443.925	5859.744	1925.234	4813.084	3369.159	10672.83	0.962617	0.970257	0.962617	0.976624
15.7	1444.511	5861.417	1926.015	4815.037	3370.526	10676.45	0.963007	0.970587	0.963007	0.976903
15.75	1445.091	5863.07	1926.787	4816.969	3371.878	10680.04	0.963394	0.970913	0.963394	0.977178
15.8	1445.664	5864.703	1927.552	4818.88	3373.216	10683.58	0.963776	0.971235	0.963776	0.977451
15.85	1446.232	5866.317	1928.309	4820.772	3374.54	10687.09	0.964154	0.971554	0.964154	0.97772
15.9	1446.793	5867.912	1929.058	4822.644	3375.851	10690.56	0.964529	0.971869	0.964529	0.977985
15.95	1447.349	5869.488	1929.799	4824.497	3377.148	10693.98	0.964899	0.97218	0.964899	0.978248
16	1447.899	5871.044	1930.532	4826.33	3378.431	10697.37	0.965266	0.972489	0.965266	0.978507
16.05	1448.443	5872.583	1931.258	4828.144	3379.701	10700.73	0.965629	0.972793	0.965629	0.978764
16.1	1448.982	5874.103	1931.976	4829.939	3380.957	10704.04	0.965988	0.973095	0.965988	0.979017
16.15	1449.515	5875.604	1932.686	4831.715	3382.201	10707.32	0.966343	0.973393	0.966343	0.979267
16.2	1450.042	5877.088	1933.389	4833.473	3383.431	10710.56	0.966695	0.973687	0.966695	0.979515
16.25	1450.564	5878.554	1934.085	4835.212	3384.649	10713.77	0.967042	0.973979	0.967042	0.979759
16.3	1451.08	5880.003	1934.774	4836.934	3385.854	10716.94	0.967387	0.974267	0.967387	0.98
16.35	1451.591	5881.434	1935.455	4838.637	3387.046	10720.07	0.967727	0.974552	0.967727	0.980239
16.4	1452.097	5882.849	1936.129	4840.322	3388.226	10723.17	0.968064	0.974834	0.968064	0.980475
16.45	1452.597	5884.246	1936.796	4841.99	3389.393	10726.24	0.968398	0.975112	0.968398	0.980708
16.5	1453.092	5885.627	1937.456	4843.641	3390.549	10729.27	0.968728	0.975388	0.968728	0.980938
16.55	1453.582	5886.991	1938.11	4845.274	3391.692	10732.26	0.969055	0.97566	0.969055	0.981165
16.6	1454.067	5888.339	1938.756	4846.89	3392.823	10735.23	0.969378	0.97593	0.969378	0.98139
16.65	1454.547	5889.671	1939.396	4848.489	3393.943	10738.16	0.969698	0.976196	0.969698	0.981612
16.7	1455.022	5890.987	1940.029	4850.072	3395.05	10741.06	0.970014	0.97646	0.970014	0.981831
16.75	1455.491	5892.287	1940.655	4851.638	3396.147	10743.93	0.970328	0.97672	0.970328	0.982048
16.8	1455.956	5893.572	1941.275	4853.188	3397.231	10746.76	0.970638	0.976978	0.970638	0.982262
16.85	1456.416	5894.842	1941.888	4854.721	3398.305	10749.56	0.970944	0.977233	0.970944	0.982474
16.9	1456.872	5896.096	1942.495	4856.239	3399.367	10752.33	0.971248	0.977485	0.971248	0.982683
16.95	1457.322	5897.336	1943.096	4857.74	3400.418	10755.08	0.971548	0.977734	0.971548	0.982889
17	1457.768	5898.56	1943.69	4859.226	3401.458	10757.79	0.971845	0.977981	0.971845	0.983093
17.05	1458.209	5899.77	1944.279	4860.696	3402.488	10760.47	0.972139	0.978224	0.972139	0.983295
17.1	1458.645	5900.966	1944.861	4862.152	3403.506	10763.12	0.97243	0.978465	0.97243	0.983494
17.15	1459.077	5902.147	1945.437	4863.591	3404.514	10765.74	0.972718	0.978703	0.972718	0.983691
17.2	1459.505	5903.314	1946.007	4865.016	3405.511	10768.33	0.973003	0.978939	0.973003	0.983886
17.25	1459.928	5904.468	1946.57	4866.426	3406.498	10770.89	0.973285	0.979172	0.973285	0.984078
17.3	1460.346	5905.607	1947.129	4867.821	3407.475	10773.43	0.973564	0.979403	0.973564	0.984268

17.35	1460.761	5906.733	1947.681	4869.202	3408.441	10775.94	0.97384	0.97963	0.97384	0.984456
17.4	1461.17	5907.846	1948.227	4870.568	3409.398	10778.41	0.974114	0.979856	0.974114	0.984641
17.45	1461.576	5908.945	1948.768	4871.92	3410.344	10780.86	0.974384	0.980079	0.974384	0.984824
17.5	1461.977	5910.031	1949.303	4873.258	3411.281	10783.29	0.974652	0.980299	0.974652	0.985005
17.55	1462.375	5911.104	1949.833	4874.582	3412.207	10785.69	0.974916	0.980517	0.974916	0.985184
17.6	1462.768	5912.165	1950.357	4875.892	3413.124	10788.06	0.975178	0.980732	0.975178	0.985361
17.65	1463.156	5913.212	1950.875	4877.188	3414.032	10790.4	0.975438	0.980946	0.975438	0.985535
17.7	1463.541	5914.248	1951.388	4878.471	3414.93	10792.72	0.975694	0.981156	0.975694	0.985708
17.75	1463.922	5915.271	1951.896	4879.74	3415.818	10795.01	0.975948	0.981365	0.975948	0.985878
17.8	1464.299	5916.281	1952.399	4880.996	3416.698	10797.28	0.976199	0.981571	0.976199	0.986047
17.85	1464.672	5917.28	1952.896	4882.239	3417.568	10799.52	0.976448	0.981774	0.976448	0.986213
17.9	1465.041	5918.267	1953.388	4883.469	3418.429	10801.74	0.976694	0.981976	0.976694	0.986378
17.95	1465.406	5919.242	1953.875	4884.687	3419.281	10803.93	0.976937	0.982175	0.976937	0.98654
18	1465.767	5920.205	1954.356	4885.891	3420.124	10806.1	0.977178	0.982372	0.977178	0.986701
18.05	1466.125	5921.157	1954.833	4887.083	3420.958	10808.24	0.977417	0.982567	0.977417	0.986859
18.1	1466.479	5922.097	1955.305	4888.262	3421.784	10810.36	0.977652	0.98276	0.977652	0.987016
18.15	1466.829	5923.026	1955.772	4889.43	3422.601	10812.46	0.977886	0.982951	0.977886	0.987171
18.2	1467.175	5923.945	1956.234	4890.584	3423.409	10814.53	0.978117	0.983139	0.978117	0.987324
18.25	1467.518	5924.852	1956.691	4891.727	3424.209	10816.58	0.978345	0.983325	0.978345	0.987475
18.3	1467.858	5925.748	1957.143	4892.858	3425.001	10818.61	0.978572	0.98351	0.978572	0.987625
18.35	1468.193	5926.634	1957.591	4893.977	3425.784	10820.61	0.978795	0.983692	0.978795	0.987772
18.4	1468.525	5927.509	1958.034	4895.085	3426.559	10822.59	0.979017	0.983872	0.979017	0.987918
18.45	1468.854	5928.374	1958.472	4896.181	3427.327	10824.55	0.979236	0.98405	0.979236	0.988062
18.5	1469.18	5929.228	1958.906	4897.265	3428.086	10826.49	0.979453	0.984227	0.979453	0.988205
18.55	1469.501	5930.072	1959.335	4898.338	3428.837	10828.41	0.979668	0.984401	0.979668	0.988345
18.6	1469.82	5930.907	1959.76	4899.4	3429.58	10830.31	0.97988	0.984573	0.97988	0.988484
18.65	1470.135	5931.731	1960.18	4900.451	3430.316	10832.18	0.98009	0.984744	0.98009	0.988622
18.7	1470.447	5932.545	1960.596	4901.491	3431.043	10834.04	0.980298	0.984912	0.980298	0.988758
18.75	1470.756	5933.35	1961.008	4902.52	3431.764	10835.87	0.980504	0.985079	0.980504	0.988892
18.8	1471.061	5934.145	1961.415	4903.538	3432.476	10837.68	0.980708	0.985244	0.980708	0.989024
18.85	1471.364	5934.93	1961.818	4904.545	3433.182	10839.48	0.980909	0.985407	0.980909	0.989155
18.9	1471.663	5935.706	1962.217	4905.542	3433.88	10841.25	0.981108	0.985568	0.981108	0.989284
18.95	1471.959	5936.473	1962.612	4906.529	3434.57	10843	0.981306	0.985727	0.981306	0.989412
19	1472.252	5937.231	1963.002	4907.505	3435.254	10844.74	0.981501	0.985885	0.981501	0.989539
19.05	1472.541	5937.98	1963.389	4908.471	3435.93	10846.45	0.981694	0.986041	0.981694	0.989663
19.1	1472.828	5938.72	1963.771	4909.428	3436.599	10848.15	0.981885	0.986195	0.981885	0.989787
19.15	1473.112	5939.451	1964.149	4910.374	3437.262	10849.82	0.982075	0.986348	0.982075	0.989908
19.2	1473.393	5940.173	1964.524	4911.31	3437.917	10851.48	0.982262	0.986498	0.982262	0.990029
19.25	1473.671	5940.887	1964.894	4912.236	3438.565	10853.12	0.982447	0.986648	0.982447	0.990148
19.3	1473.946	5941.592	1965.261	4913.153	3439.207	10854.74	0.982631	0.986795	0.982631	0.990265
19.35	1474.218	5942.288	1965.624	4914.06	3439.842	10856.35	0.982812	0.986941	0.982812	0.990381
19.4	1474.487	5942.977	1965.983	4914.958	3440.47	10857.93	0.982992	0.987085	0.982992	0.990496
19.45	1474.754	5943.657	1966.338	4915.846	3441.092	10859.5	0.983169	0.987228	0.983169	0.99061
19.5	1475.018	5944.329	1966.69	4916.725	3441.707	10861.05	0.983345	0.987369	0.983345	0.990722
19.55	1475.278	5944.993	1967.038	4917.595	3442.316	10862.59	0.983519	0.987508	0.983519	0.990832
19.6	1475.537	5945.649	1967.382	4918.455	3442.919	10864.1	0.983691	0.987646	0.983691	0.990942
19.65	1475.792	5946.298	1967.723	4919.307	3443.515	10865.6	0.983861	0.987782	0.983861	0.99105
19.7	1476.045	5946.938	1968.06	4920.15	3444.105	10867.09	0.98403	0.987917	0.98403	0.991156
19.75	1476.295	5947.571	1968.394	4920.984	3444.689	10868.56	0.984197	0.98805	0.984197	0.991262
19.8	1476.543	5948.197	1968.724	4921.809	3445.267	10870.01	0.984362	0.988182	0.984362	0.991366
19.85	1476.788	5948.814	1969.05	4922.626	3445.838	10871.44	0.984525	0.988313	0.984525	0.991469
19.9	1477.03	5949.425	1969.374	4923.434	3446.404	10872.86	0.984687	0.988442	0.984687	0.991571
19.95	1477.27	5950.028	1969.694	4924.234	3446.964	10874.26	0.984847	0.988569	0.984847	0.991671
20	1477.508	5950.624	1970.01	4925.025	3447.518	10875.65	0.985005	0.988695	0.985005	0.991771
20.05	1477.743	5951.213	1970.323	4925.809	3448.066	10877.02	0.985162	0.98882	0.985162	0.991869
20.1	1477.975	5951.795	1970.633	4926.584	3448.608	10878.38	0.985317	0.988944	0.985317	0.991966
20.15	1478.205	5952.37	1970.94	4927.35	3449.145	10879.72	0.98547	0.989066	0.98547	0.992062
20.2	1478.433	5952.938	1971.244	4928.109	3449.677	10881.05	0.985622	0.989186	0.985622	0.992156
20.25	1478.658	5953.5	1971.544	4928.86	3450.202	10882.36	0.985772	0.989305	0.985772	0.99225
20.3	1478.881	5954.055	1971.841	4929.603	3450.722	10883.66	0.985921	0.989423	0.985921	0.992342
20.35	1479.102	5954.603	1972.135	4930.339	3451.237	10884.94	0.986068	0.98954	0.986068	0.992434
20.4	1479.32	5955.144	1972.426	4931.066	3451.746	10886.21	0.986213	0.989655	0.986213	0.992524
20.45	1479.536	5955.679	1972.714	4931.786	3452.25	10887.47	0.986357	0.98977	0.986357	0.992613
20.5	1479.75	5956.208	1972.999	4932.499	3452.749	10888.71	0.9865	0.989882	0.9865	0.992701
20.55	1479.961	5956.73	1973.282	4933.204	3453.243	10889.93	0.986641	0.989994	0.986641	0.992788
20.6	1480.17	5957.246	1973.561	4933.901	3453.731	10891.15	0.98678	0.990104	0.98678	0.992874
20.65	1480.378	5957.756	1973.837	4934.592	3454.214	10892.35	0.986918	0.990213	0.986918	0.992959
20.7	1480.583	5958.26	1974.11	4935.275	3454.693	10893.54	0.987055	0.990321	0.987055	0.993043
20.75	1480.785	5958.758	1974.38	4935.951	3455.166	10894.71	0.98719	0.990428	0.98719	0.993126
20.8	1480.986	5959.25	1974.648	4936.62	3455.634	10895.87	0.987324	0.990534	0.987324	0.993208
20.85	1481.185	5959.736	1974.913	4937.282	3456.097	10897.02	0.987456	0.990638	0.987456	0.993289
20.9	1481.381	5960.216	1975.175	4937.937	3456.556	10898.15	0.987587	0.990741	0.987587	0.993369
20.95	1481.576	5960.691	1975.434	4938.585	3457.01	10899.28	0.987717	0.990843	0.987717	0.993449
21	1481.768	5961.16	1975.691	4939.227	3457.459	10900.39	0.987845	0.990944	0.987845	0.993527
21.05	1481.959	5961.623	1975.945	4939.862	3457.903	10901.48	0.987972	0.991044	0.987972	0.993604
21.1	1482.147	5962.081	1976.196	4940.49	3458.343	10902.57	0.988098	0.991143	0.988098	0.99368
21.15	1482.333	5962.533	1976.445	4941.112	3458.778	10903.64	0.988222	0.99124	0.988222	0.993756
21.2	1482.518	5962.98	1976.691	4941.727	3459.209	10904.71	0.988345	0.991337	0.988345	0.99383
21.25	1482.701	5963.422	1976.934	4942.335	3459.635	10905.76	0.988467	0.991432	0.988467	0.993904
21.3	1482.881	5963.858	1977.175	4942.938	3460.056	10906.8	0.988588	0.991527	0.988588	0.993976
21.35	1483.06	5964.289	1977.413	4943.534	3460.474	10907.82	0.988707	0.99162	0.988707	0.994048
21.4	1483.237	5964.715	1977.649	4944.123	3460.886	10908.84	0.988825	0.991713	0.988825	0.994119
21.45	1483.412	5965.136	1977.883	4944.707	3461.295	10909.84	0.988941	0.991804	0.988941	0.994189
21.5	1483.585	5965.552	1978.114	4945.285	3461.699	10910.84	0.989057	0.991894	0.989057	0.994259
21.55	1483.757	5965.963	1978.342	4945.856	3462.099	10911.82	0.989171	0.991984	0.989171	0.994327
21.6	1483.927	5966.369	1978.569	4946.422	3462.495	10912.79	0.989284	0.992072	0.989284	0.994395
21.65	1484.094	5966.77	1978.793	4946.981	3462.887	10913.75	0.989396	0.992159	0.989396	0.994462

21.7	1484.261	5967.166	1979.014	4947.535	3463.275	10914.7	0.989507	0.992246	0.989507	0.994528
21.75	1484.425	5967.558	1979.233	4948.083	3463.658	10915.64	0.989617	0.992331	0.989617	0.994593
21.8	1484.588	5967.945	1979.45	4948.625	3464.038	10916.57	0.989725	0.992415	0.989725	0.994658
21.85	1484.749	5968.327	1979.665	4949.162	3464.413	10917.49	0.989832	0.992499	0.989832	0.994721
21.9	1484.908	5968.705	1979.877	4949.693	3464.785	10918.4	0.989939	0.992582	0.989939	0.994784
21.95	1485.066	5969.079	1980.087	4950.218	3465.153	10919.3	0.990044	0.992663	0.990044	0.994846
22	1485.222	5969.447	1980.295	4950.738	3465.517	10920.19	0.990148	0.992744	0.990148	0.994908
22.05	1485.376	5969.812	1980.501	4951.253	3465.877	10921.06	0.990251	0.992824	0.990251	0.994969
22.1	1485.529	5970.172	1980.705	4951.762	3466.234	10921.93	0.990352	0.992903	0.990352	0.995029
22.15	1485.68	5970.528	1980.906	4952.266	3466.586	10922.79	0.990453	0.992981	0.990453	0.995088
22.2	1485.829	5970.879	1981.106	4952.765	3466.935	10923.64	0.990553	0.993059	0.990553	0.995147
22.25	1485.977	5971.227	1981.303	4953.258	3467.281	10924.48	0.990652	0.993135	0.990652	0.995204
22.3	1486.124	5971.57	1981.499	4953.746	3467.622	10925.32	0.990749	0.993211	0.990749	0.995262
22.35	1486.269	5971.909	1981.692	4954.229	3467.961	10926.14	0.990846	0.993285	0.990846	0.995318
22.4	1486.412	5972.244	1981.883	4954.707	3468.295	10926.95	0.990941	0.993359	0.990941	0.995374
22.45	1486.554	5972.575	1982.072	4955.181	3468.626	10927.76	0.991036	0.993432	0.991036	0.995429
22.5	1486.695	5972.902	1982.26	4955.649	3468.954	10928.55	0.99113	0.993505	0.99113	0.995484
22.55	1486.834	5973.226	1982.445	4956.112	3469.278	10929.34	0.991222	0.993576	0.991222	0.995538
22.6	1486.971	5973.545	1982.628	4956.57	3469.599	10930.12	0.991314	0.993647	0.991314	0.995591
22.65	1487.107	5973.86	1982.81	4957.024	3469.917	10930.88	0.991405	0.993717	0.991405	0.995643
22.7	1487.242	5974.172	1982.989	4957.473	3470.231	10931.65	0.991495	0.993786	0.991495	0.995695
22.75	1487.375	5974.48	1983.167	4957.917	3470.542	10932.4	0.991583	0.993854	0.991583	0.995747
22.8	1487.507	5974.785	1983.343	4958.357	3470.85	10933.14	0.991671	0.993922	0.991671	0.995797
22.85	1487.638	5975.086	1983.517	4958.792	3471.154	10933.88	0.991758	0.993989	0.991758	0.995848
22.9	1487.767	5975.383	1983.689	4959.222	3471.455	10934.6	0.991844	0.994055	0.991844	0.995897
22.95	1487.894	5975.676	1983.859	4959.648	3471.754	10935.32	0.99193	0.99412	0.99193	0.995946
23	1488.021	5975.967	1984.028	4960.069	3472.049	10936.04	0.992014	0.994185	0.992014	0.995994
23.05	1488.146	5976.253	1984.195	4960.487	3472.341	10936.74	0.992097	0.994249	0.992097	0.996042
23.1	1488.27	5976.536	1984.36	4960.899	3472.63	10937.44	0.99218	0.994312	0.99218	0.996089
23.15	1488.392	5976.816	1984.523	4961.308	3472.915	10938.12	0.992262	0.994375	0.992262	0.996136
23.2	1488.514	5977.093	1984.685	4961.712	3473.198	10938.8	0.992342	0.994437	0.992342	0.996182
23.25	1488.634	5977.366	1984.845	4962.112	3473.478	10939.48	0.992422	0.994498	0.992422	0.996228
23.3	1488.752	5977.636	1985.003	4962.508	3473.755	10940.14	0.992501	0.994559	0.992501	0.996273
23.35	1488.87	5977.903	1985.16	4962.899	3474.029	10940.8	0.99258	0.994618	0.99258	0.996317
23.4	1488.986	5978.167	1985.315	4963.287	3474.301	10941.45	0.992657	0.994678	0.992657	0.996361
23.45	1489.101	5978.427	1985.468	4963.67	3474.569	10942.1	0.992734	0.994736	0.992734	0.996404
23.5	1489.215	5978.684	1985.62	4964.05	3474.835	10942.73	0.99281	0.994794	0.99281	0.996447
23.55	1489.328	5978.939	1985.77	4964.425	3475.098	10943.36	0.992885	0.994851	0.992885	0.99649
23.6	1489.439	5979.19	1985.919	4964.797	3475.358	10943.99	0.992959	0.994908	0.992959	0.996532
23.65	1489.549	5979.438	1986.066	4965.164	3475.615	10944.6	0.993033	0.994964	0.993033	0.996573
23.7	1489.659	5979.683	1986.211	4965.528	3475.87	10945.21	0.993106	0.995019	0.993106	0.996614
23.75	1489.767	5979.926	1986.355	4965.888	3476.122	10945.81	0.993178	0.995074	0.993178	0.996654
23.8	1489.873	5980.165	1986.498	4966.245	3476.371	10946.41	0.993249	0.995128	0.993249	0.996694
23.85	1489.979	5980.402	1986.639	4966.597	3476.618	10947	0.993319	0.995182	0.993319	0.996734
23.9	1490.084	5980.635	1986.778	4966.946	3476.862	10947.58	0.993389	0.995235	0.993389	0.996773
23.95	1490.187	5980.866	1986.917	4967.291	3477.104	10948.16	0.993458	0.995287	0.993458	0.996811
24	1490.29	5981.095	1987.053	4967.633	3477.343	10948.73	0.993527	0.995339	0.993527	0.996849
24.05	1490.391	5981.32	1987.188	4967.971	3477.58	10949.29	0.993594	0.99539	0.993594	0.996887
24.1	1490.492	5981.543	1987.322	4968.306	3477.814	10949.85	0.993661	0.995441	0.993661	0.996924
24.15	1490.591	5981.763	1987.455	4968.637	3478.046	10950.4	0.993727	0.995491	0.993727	0.996961
24.2	1490.689	5981.981	1987.586	4968.964	3478.275	10950.94	0.993793	0.99554	0.993793	0.996997
24.25	1490.787	5982.196	1987.715	4969.288	3478.502	10951.48	0.993858	0.995589	0.993858	0.997033
24.3	1490.883	5982.408	1987.844	4969.609	3478.727	10952.02	0.993922	0.995638	0.993922	0.997068
24.35	1490.978	5982.618	1987.971	4969.927	3478.949	10952.54	0.993985	0.995686	0.993985	0.997103
24.4	1491.072	5982.825	1988.096	4970.241	3479.169	10953.07	0.994048	0.995733	0.994048	0.997138
24.45	1491.166	5983.03	1988.221	4970.552	3479.386	10953.58	0.99411	0.99578	0.99411	0.997172
24.5	1491.258	5983.233	1988.344	4970.859	3479.602	10954.09	0.994172	0.995827	0.994172	0.997205
24.55	1491.349	5983.433	1988.465	4971.164	3479.815	10954.6	0.994233	0.995872	0.994233	0.997239
24.6	1491.439	5983.63	1988.586	4971.465	3480.025	10955.09	0.994293	0.995918	0.994293	0.997272
24.65	1491.529	5983.825	1988.705	4971.763	3480.234	10955.59	0.994353	0.995963	0.994353	0.997304
24.7	1491.617	5984.018	1988.823	4972.058	3480.44	10956.08	0.994412	0.996007	0.994412	0.997336
24.75	1491.705	5984.209	1988.94	4972.35	3480.645	10956.56	0.99447	0.996051	0.99447	0.997368
24.8	1491.792	5984.397	1989.055	4972.639	3480.847	10957.04	0.994528	0.996094	0.994528	0.9974
24.85	1491.877	5984.583	1989.17	4972.924	3481.047	10957.51	0.994585	0.996137	0.994585	0.997431
24.9	1491.962	5984.767	1989.283	4973.207	3481.245	10957.97	0.994641	0.996179	0.994641	0.997461
24.95	1492.046	5984.949	1989.395	4973.487	3481.441	10958.44	0.994697	0.996221	0.994697	0.997492
25	1492.129	5985.129	1989.506	4973.764	3481.635	10958.89	0.994753	0.996263	0.994753	0.997521
25.05	1492.211	5985.306	1989.615	4974.038	3481.827	10959.34	0.994808	0.996304	0.994808	0.997551
25.1	1492.293	5985.481	1989.724	4974.309	3482.016	10959.79	0.994862	0.996345	0.994862	0.99758
25.15	1492.373	5985.654	1989.831	4974.577	3482.204	10960.23	0.994915	0.996385	0.994915	0.997609
25.2	1492.453	5985.826	1989.937	4974.843	3482.39	10960.67	0.994969	0.996424	0.994969	0.997638
25.25	1492.532	5985.995	1990.042	4975.106	3482.574	10961.1	0.995021	0.996464	0.995021	0.997666
25.3	1492.61	5986.162	1990.146	4975.366	3482.756	10961.53	0.995073	0.996503	0.995073	0.997694
25.35	1492.687	5986.327	1990.249	4975.623	3482.936	10961.95	0.995125	0.996541	0.995125	0.997721
25.4	1492.763	5986.49	1990.351	4975.878	3483.114	10962.37	0.995176	0.996579	0.995176	0.997748
25.45	1492.839	5986.651	1990.452	4976.13	3483.291	10962.78	0.995226	0.996616	0.995226	0.997775
25.5	1492.914	5986.81	1990.552	4976.379	3483.465	10963.19	0.995276	0.996654	0.995276	0.997802
25.55	1492.988	5986.968	1990.65	4976.626	3483.638	10963.59	0.995325	0.99669	0.995325	0.997828
25.6	1493.061	5987.123	1990.748	4976.87	3483.809	10963.99	0.995374	0.996727	0.995374	0.997854
25.65	1493.133	5987.277	1990.845	4977.112	3483.978	10964.39	0.995422	0.996763	0.995422	0.997879
25.7	1493.205	5987.428	1990.94	4977.351	3484.145	10964.78	0.99547	0.996798	0.99547	0.997905
25.75	1493.276	5987.578	1991.035	4977.587	3484.311	10965.17	0.995517	0.996833	0.995517	0.99793
25.8	1493.346	5987.727	1991.129	4977.821	3484.475	10965.55	0.995564	0.996868	0.995564	0.997954
25.85	1493.416	5987.873	1991.221	4978.053	3484.637	10965.93	0.995611	0.996902	0.995611	0.997979
25.9	1493.485	5988.018	1991.313	4978.282	3484.797	10966.3	0.995656	0.996936	0.995656	0.998003
25.95	1493.553	5988.161	1991.404	4978.509	3484.956	10966.67	0.995702	0.99697	0.995702	0.998027
26	1493.62	5988.302	1991.493	4978.733	3485.113	10967.04	0.995747	0.997003	0.995747	0.99805

26.05	1493.687	5988.441	1991.582	4978.956	3485.269	10967.4	0.995791	0.997036	0.995791	0.998074
26.1	1493.753	5988.579	1991.67	4979.175	3485.423	10967.75	0.995835	0.997069	0.995835	0.998097
26.15	1493.818	5988.715	1991.757	4979.393	3485.575	10968.11	0.995879	0.997101	0.995879	0.998119
26.2	1493.882	5988.85	1991.843	4979.608	3485.726	10968.46	0.995922	0.997133	0.995922	0.998142
26.25	1493.946	5988.983	1991.929	4979.821	3485.875	10968.8	0.995964	0.997164	0.995964	0.998164
26.3	1494.01	5989.114	1992.013	4980.032	3486.022	10969.15	0.996006	0.997195	0.996006	0.998186
26.35	1494.072	5989.244	1992.096	4980.241	3486.168	10969.48	0.996048	0.997226	0.996048	0.998207
26.4	1494.134	5989.373	1992.179	4980.447	3486.313	10969.82	0.996089	0.997256	0.996089	0.998229
26.45	1494.195	5989.499	1992.26	4980.651	3486.456	10970.15	0.99613	0.997286	0.99613	0.99825
26.5	1494.256	5989.625	1992.341	4980.853	3486.597	10970.48	0.996171	0.997316	0.996171	0.998271
26.55	1494.316	5989.748	1992.421	4981.053	3486.737	10970.8	0.996211	0.997346	0.996211	0.998291
26.6	1494.375	5989.871	1992.5	4981.251	3486.876	10971.12	0.99625	0.997375	0.99625	0.998312
26.65	1494.434	5989.992	1992.579	4981.447	3487.013	10971.44	0.996289	0.997403	0.996289	0.998332
26.7	1494.492	5990.111	1992.656	4981.641	3487.149	10971.75	0.996328	0.997432	0.996328	0.998352
26.75	1494.55	5990.229	1992.733	4981.833	3487.283	10972.06	0.996367	0.99746	0.996367	0.998371
26.8	1494.607	5990.345	1992.809	4982.022	3487.416	10972.37	0.996404	0.997488	0.996404	0.998391
26.85	1494.663	5990.461	1992.884	4982.21	3487.547	10972.67	0.996442	0.997516	0.996442	0.99841
26.9	1494.719	5990.574	1992.958	4982.396	3487.677	10972.97	0.996479	0.997543	0.996479	0.998429
26.95	1494.774	5990.687	1993.032	4982.58	3487.806	10973.27	0.996516	0.99757	0.996516	0.998448
27	1494.829	5990.798	1993.105	4982.762	3487.933	10973.56	0.996552	0.997596	0.996552	0.998466
27.05	1494.883	5990.908	1993.177	4982.942	3488.059	10973.85	0.996588	0.997623	0.996588	0.998485
27.1	1494.936	5991.016	1993.248	4983.12	3488.184	10974.14	0.996624	0.997649	0.996624	0.998503
27.15	1494.989	5991.123	1993.319	4983.296	3488.307	10974.42	0.996659	0.997674	0.996659	0.998521
27.2	1495.041	5991.229	1993.388	4983.471	3488.43	10974.7	0.996694	0.9977	0.996694	0.998538
27.25	1495.093	5991.334	1993.457	4983.643	3488.55	10974.98	0.996729	0.997725	0.996729	0.998556
27.3	1495.144	5991.437	1993.526	4983.814	3488.67	10975.25	0.996763	0.99775	0.996763	0.998573
27.35	1495.195	5991.539	1993.593	4983.983	3488.788	10975.52	0.996797	0.997775	0.996797	0.99859
27.4	1495.245	5991.64	1993.66	4984.151	3488.905	10975.79	0.99683	0.997799	0.99683	0.998607
27.45	1495.295	5991.74	1993.727	4984.316	3489.021	10976.06	0.996863	0.997823	0.996863	0.998623
27.5	1495.344	5991.838	1993.792	4984.48	3489.136	10976.32	0.996896	0.997847	0.996896	0.99864
27.55	1495.393	5991.936	1993.857	4984.642	3489.249	10976.58	0.996928	0.997871	0.996928	0.998656
27.6	1495.441	5992.032	1993.921	4984.803	3489.362	10976.83	0.99696	0.997894	0.99696	0.998672
27.65	1495.488	5992.127	1993.985	4984.961	3489.473	10977.09	0.996992	0.997917	0.996992	0.998688
27.7	1495.536	5992.221	1994.047	4985.118	3489.583	10977.34	0.997024	0.99794	0.997024	0.998703
27.75	1495.582	5992.314	1994.11	4985.274	3489.692	10977.59	0.997055	0.997962	0.997055	0.998719
27.8	1495.628	5992.405	1994.171	4985.428	3489.799	10977.83	0.997086	0.997985	0.997086	0.998734
27.85	1495.674	5992.496	1994.232	4985.58	3489.906	10978.08	0.997116	0.998007	0.997116	0.998749
27.9	1495.719	5992.586	1994.292	4985.73	3490.011	10978.32	0.997146	0.998029	0.997146	0.998764
27.95	1495.764	5992.674	1994.352	4985.879	3490.116	10978.55	0.997176	0.99805	0.997176	0.998779
28	1495.808	5992.761	1994.411	4986.027	3490.219	10978.79	0.997205	0.998072	0.997205	0.998794
28.05	1495.852	5992.848	1994.469	4986.173	3490.321	10979.02	0.997235	0.998093	0.997235	0.998808
28.1	1495.895	5992.933	1994.527	4986.317	3490.422	10979.25	0.997263	0.998114	0.997263	0.998822
28.15	1495.938	5993.017	1994.584	4986.46	3490.522	10979.48	0.997292	0.998134	0.997292	0.998836
28.2	1495.981	5993.101	1994.641	4986.602	3490.621	10979.7	0.99732	0.998155	0.99732	0.99885
28.25	1496.023	5993.183	1994.697	4986.742	3490.719	10979.92	0.997348	0.998175	0.997348	0.998864
28.3	1496.064	5993.264	1994.752	4986.88	3490.816	10980.14	0.997376	0.998195	0.997376	0.998877
28.35	1496.105	5993.345	1994.807	4987.017	3490.912	10980.36	0.997403	0.998215	0.997403	0.998891
28.4	1496.146	5993.424	1994.861	4987.153	3491.007	10980.58	0.997431	0.998234	0.997431	0.998904
28.45	1496.186	5993.502	1994.915	4987.287	3491.101	10980.79	0.997457	0.998254	0.997457	0.998917
28.5	1496.226	5993.58	1994.968	4987.42	3491.194	10981	0.997484	0.998273	0.997484	0.99893
28.55	1496.265	5993.657	1995.021	4987.551	3491.286	10981.21	0.99751	0.998292	0.99751	0.998943
28.6	1496.304	5993.732	1995.073	4987.681	3491.377	10981.41	0.997536	0.99831	0.997536	0.998955
28.65	1496.343	5993.807	1995.124	4987.81	3491.467	10981.62	0.997562	0.998329	0.997562	0.998968
28.7	1496.381	5993.881	1995.175	4987.937	3491.556	10981.82	0.997587	0.998347	0.997587	0.99898
28.75	1496.419	5993.954	1995.225	4988.063	3491.644	10982.02	0.997613	0.998365	0.997613	0.998992
28.8	1496.456	5994.026	1995.275	4988.188	3491.732	10982.21	0.997638	0.998383	0.997638	0.999004
28.85	1496.493	5994.097	1995.325	4988.311	3491.818	10982.41	0.997662	0.998401	0.997662	0.999016
28.9	1496.53	5994.168	1995.373	4988.433	3491.903	10982.6	0.997687	0.998418	0.997687	0.999028
28.95	1496.566	5994.237	1995.422	4988.554	3491.988	10982.79	0.997711	0.998436	0.997711	0.99904
29	1496.602	5994.306	1995.47	4988.674	3492.072	10982.98	0.997735	0.998453	0.997735	0.999051
29.05	1496.638	5994.374	1995.517	4988.792	3492.154	10983.17	0.997758	0.99847	0.997758	0.999062
29.1	1496.673	5994.441	1995.564	4988.909	3492.236	10983.35	0.997782	0.998486	0.997782	0.999073
29.15	1496.708	5994.507	1995.61	4989.025	3492.318	10983.53	0.997805	0.998503	0.997805	0.999085
29.2	1496.742	5994.573	1995.656	4989.14	3492.398	10983.71	0.997828	0.998519	0.997828	0.999095
29.25	1496.776	5994.638	1995.701	4989.253	3492.477	10983.89	0.997851	0.998536	0.997851	0.999106
29.3	1496.81	5994.702	1995.746	4989.365	3492.556	10984.07	0.997873	0.998552	0.997873	0.999117
29.35	1496.843	5994.765	1995.791	4989.476	3492.633	10984.24	0.997895	0.998567	0.997895	0.999127
29.4	1496.876	5994.827	1995.835	4989.586	3492.71	10984.41	0.997917	0.998583	0.997917	0.999138
29.45	1496.909	5994.889	1995.878	4989.695	3492.787	10984.58	0.997939	0.998599	0.997939	0.999148
29.5	1496.941	5994.95	1995.921	4989.803	3492.862	10984.75	0.997961	0.998614	0.997961	0.999158
29.55	1496.973	5995.01	1995.964	4989.909	3492.936	10984.92	0.997982	0.998629	0.997982	0.999168
29.6	1497.004	5995.07	1996.006	4990.015	3493.01	10985.08	0.998003	0.998644	0.998003	0.999178
29.65	1497.036	5995.128	1996.048	4990.119	3493.083	10985.25	0.998024	0.998659	0.998024	0.999188
29.7	1497.067	5995.187	1996.089	4990.222	3493.155	10985.41	0.998044	0.998674	0.998044	0.999198
29.75	1497.097	5995.244	1996.13	4990.324	3493.227	10985.57	0.998065	0.998688	0.998065	0.999207
29.8	1497.128	5995.301	1996.17	4990.425	3493.298	10985.73	0.998085	0.998702	0.998085	0.999217
29.85	1497.158	5995.357	1996.21	4990.525	3493.368	10985.88	0.998105	0.998717	0.998105	0.999226
29.9	1497.187	5995.412	1996.25	4990.624	3493.437	10986.04	0.998125	0.998731	0.998125	0.999235
29.95	1497.217	5995.467	1996.289	4990.722	3493.506	10986.19	0.998144	0.998744	0.998144	0.999244
30	1497.246	5995.521	1996.328	4990.819	3493.573	10986.34	0.998164	0.998758	0.998164	0.999253
