# Supplementary material for: Reorganization of synchronous and asynchronous release in developing hippocampal autaptic neurons of mice
Source: Physiol Rep. 2026 Aug 17;14(16):e71062. doi: 10.14814/phy2.71062 (PMC13481710; doi:10.14814/phy2.71062)

Uchino et al.
Supplementary
Figure. S1

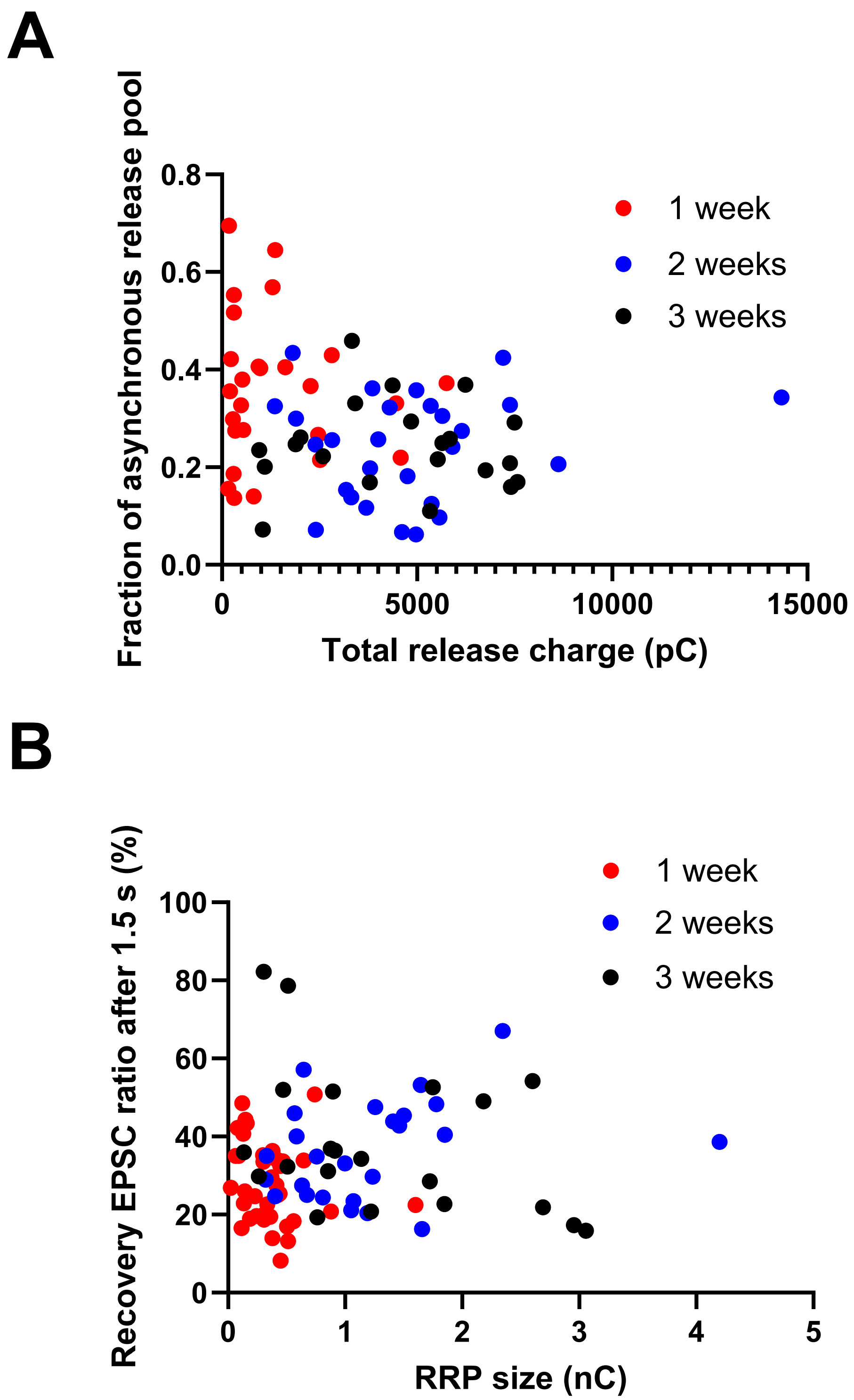

Supplement: Supplementary file 4 [file PHY2-14-e71062-s004.pdf]
